# A CFD and Experimental Investigation of the Influence of Flow Characteristics on Spherical Agglomeration

**DOI:** 10.3390/pharmaceutics18030301

**Published:** 2026-02-27

**Authors:** Victoria R. Kitching, Kate Pitt, Bilal Ahmed, James D. Litster, Rachel M. Smith

**Affiliations:** 1 Department of Chemical and Biological Engineering, The University of Sheffield, Sheffield S1 3JD, UK; v.r.kitching@leeds.ac.uk (V.R.K.); k.pitt@sheffield.ac.uk (K.P.);; 2 CMAC (Continuous Manufacturing and Manufacturing and Advanced Crystallisation), The University of Strathclyde, Glasgow G1 1RD, UK; bilal.ahmed@strath.ac.uk

**Keywords:** spherical agglomeration, CFD, stirred tank design, impeller configuration

## Abstract

**Background/Objectives**: Spherical agglomeration is a particle size enlargement technique with promise to improve micromeritic properties of active pharmaceutical ingredients. In a spherical agglomeration process, an immiscible bridging liquid is added to suspended crystals, inducing agglomeration. Interaction between primary particles and bridging liquid is essential for agglomeration to occur, and mixing is critical as it influences flow profiles and particle suspension. Benchtop-scale stirred tanks are commonly used for spherical agglomeration research. However, there is little consistency in the tank and impeller design, resulting in limited understanding of the influence mixing has on agglomerate properties. **Methods**: To inform spherical agglomeration reactor design, four industrial standard impeller geometries promoting differing levels of radial and axial flow in the tank were tested in a 1 L stirred tank at impeller speeds ranging from 300 rpm to 600 rpm. The impeller clearance-to-vessel diameter ratio was varied between 0.18 and 0.33 to determine the influence that impeller characteristics have on spherical agglomerates. Corresponding CFD simulations were conducted in ANSYS Fluent to understand vessel flow patterns with different impeller geometries, speeds and clearances. **Results**: Experimental results suggest impellers with increased power number produce more consistent agglomerates. CFD simulations showed a clear influence of impeller clearance on particle suspension and velocity profile in the tank. **Conclusions**: Whilst experimental studies and CFD studies have been conducted for spherical agglomeration, this work provides a systematic investigation that compares both CFD and experimental analysis for industrial standard impeller geometries to understand the important, yet underexamined link between impeller characteristics and spherical agglomerate shape and size.

## 1. Introduction

Spherical agglomeration has received interest from the pharmaceutical industry as this process can transform needle-shaped crystals into dense agglomerates with increased sphericity, improving micromeritic properties such as ease of handling and flowability [1,2]. Previous work has demonstrated the ability for agglomerate porosity to be tuned [3]. This enables direct tabletting of spherical agglomerates, reducing the need for further processing, which, in turn, minimises production costs [4]. The ability for spherical agglomerates to be directly tabletted makes this process attractive to the pharmaceutical industry as oral solid dosage forms are the most common form of administration to patients, with 70% of all pharmaceutical products being in solid form [5,6].

The spherical agglomeration process begins with particles in a suspension or slurry. A commonly used starting point is antisolvent crystallisation. Here, an active pharmaceutical ingredient (API) is dissolved in a solvent and antisolvent addition results in crystal formation. Agglomeration in suspension can also be used to manufacture spherical agglomerates, where previously formed API crystals are suspended in a solvent which acts as a dispersion medium, then an immiscible bridging liquid is added, inducing agglomeration [7].

Once a slurry or suspension is achieved, the spherical agglomeration commences with the addition of an immiscible bridging liquid, inducing agglomeration. The selection of bridging liquid is an important task in determining the spherical agglomeration solvent system as it must preferentially wet the API [8]. As spherical agglomeration occurs in a ternary mixture, the selection of the liquids is extremely important for the process to work effectively, and this choice can be difficult and time-consuming [9]. Therefore, the majority of spherical agglomeration research has focussed on determining the optimal solvent system for a specific API.

Arjmandi-Tash et al. proposed two mechanisms of spherical agglomerate formation: the immersion mechanism and the distribution mechanism [10]. If the API particles are larger than the bridging liquid droplets, the distribution mechanism is expected to occur. In the distribution mechanism, the droplets coat the particles and agglomerates form due to contact between the wetted particles. In the immersion mechanism, the bridging liquid droplets are larger than the API particles. The particles immerse into the bridging liquid droplets to form agglomerates. Therefore, the immersion mechanism can theoretically control agglomerate size, with larger bridging liquid droplets resulting in larger agglomerates [10,11].

A population balance model (PBM) was developed by Ahmed et al. which incorporated the immersion and distribution mechanisms of spherical agglomeration [12]. The PBM was implemented in Siemens PSE gPROMS Formulated Products using the high-shear granulator framework as a basis, due to some mechanistic similarity between spherical agglomeration and high-shear wet granulation [2]. In the model, custom agglomeration and layering kernels are used to allow for growth due to the immersion mechanism, the distribution mechanism and coalescence. This model considers the properties of the API and solvent system, the impeller speed, the bridging liquid-to-solid ratio (BSR), the solid loading and other important parameters. However, it does not consider the influence of impeller geometry or clearance [12].

Stirred tanks are commonly used for pharmaceutical manufacturing as they have proven to be reliable at solids handling [13,14]. There is interest in progressing pharmaceutical manufacturing from batch to continuous processing. Continuous stirred tank reactors (CSTRs) are frequently used at large scale as they have improved solids handling and mixing efficiency. There is also substantial knowledge of efficient stirred tank scale-up, making them attractive for industrial scale production [14]. Common issues which arise during stirred tank scale-up include changes in mixing, particle formation, reaction sensitivity to unideal mixing, impurity formation and inefficient heat transfer leading to hotspots [15,16]. Three similarity strategies are typically utilised for scale-up to minimise deviations from the benchtop scale, they are dynamic similarity, geometric similarity and kinematic similarity. Dynamic similarity considers the forces within the tank, including Reynolds number and Froude number. Geometric similarity is achieved by keeping the same dimensional ratios of the stirred tank and the agitator. Kinematic similarity is obtained by keeping the velocity profiles in the tank similar to those obtained at smaller scales, as described by the impeller tip speed [17]. It is essential to consider all possible scale-up strategies during scale-up, to ensure critical rate processes are scaled-up as desired.

The mixing behaviour in the stirred tank will influence the contact between the API particles and the bridging liquid in the agglomerating vessel. Investigations into the influence of impeller speed on the formation of spherical agglomerates have determined that increased impeller speed reduces the size of the agglomerates [18,19]. As Equation (1) shows, increased impeller speed results in increased shear forces in the system [20].

(1) $$\dot{\gamma }=\Lambda \frac{V_{tip}}{R}$$

where $\dot{\gamma }$ is the shear (time-averaged velocity gradient), Λ is the shear rate constant, $V_{tip}$ is the impeller tip speed (m/s) and R is impeller radius (m).

Increased shear forces can result in attrition and breakage of the agglomerates, which can lead to a broad particle size distribution [21,22]. Increased impeller speed can also influence the sphericity of the agglomerates as increased shear increases the agglomeration rate constant exponentially; this will lead to faster agglomerate formation and consolidation [21,23].

Whilst multiple studies have considered the influence of impeller speed on spherical agglomeration, only one study has investigated different impeller geometries. Chen et al., 2021 tested three different impeller geometries at multiple scales [23]. In this work, the Maxblend impeller (a paddle surrounded by a grid covering approximately 60% of the vessel height) was found to require lower impeller speeds to suspend the particles compared to the single and double configuration pitched-blade impeller geometries that were also tested.

The critical impeller speed for particle suspension was proposed by Zwietering in 1958; it can be used to calculate the impeller speed at which there are no particles settled at the bottom of the tank for more than 2 s [24]. A study by Devarajulu and Loganathan determined the Zwietering constant for different impeller geometries including a Rushton turbine impeller and a pitched-blade impeller [25]. The proposed Zwietering constants were found to be dependent on the impeller clearance, liquid height and tank diameter [25]. There are limitations to the application of the Zwietering equation. In a study by Ayranci and Kresta, it was found that the influence of impeller clearance on critical impeller speed cannot be separately accounted for in the Zwietering equation and that experimental work is needed to derive accurate constants [26]. The equation for the just-suspended impeller speed is shown in Equation (2).

(2) $$N_{js}=\frac{1}{60}Sv^{0.1}{\left[\frac{g\left(\rho_{s}-\rho_{l}\right)}{\rho_{l}}\right]}^{0.45}X^{0.13}{d_{p}}^{0.2}d^{-0.85}$$

where $N_{js}$ is the critical impeller speed for particle suspension (rpm), S is the Zwietering constant, v is the kinematic viscosity (m^2^/s), g is gravitational acceleration (m/s), $\rho_{s}$ is the solid density (kg/m^3^), $\rho_{l}$ is the liquid density (kg/m^3^), X is the solid loading in the system, $d_{p}$ is the diameter of the particles (m), and d is the diameter of the impeller (m).

The influence of impeller clearance on flow characteristics is extremely important with a Rushton turbine impeller. Both experimental and CFD studies determined that at an impeller clearance-to-tank diameter (C/D) ratio below 0.3, the characteristic double-loop flow pattern that is expected from a radial impeller such as a Rushton turbine is unable to form. In these cases, the Rushton turbine operates as an axial flow impeller [27,28]. A single-loop flow pattern is thought to improve particle suspension, as double-loop flow has been shown to lead to segregation in the tank [29]. The impeller power number is an important parameter that describes the power requirements for moving the fluid in the system. Different impeller geometries have different power numbers, with radial flow impellers generally having higher power numbers than axial flow impellers [20,30].

CFD studies numerically solve mass, momentum and energy equations to simulate the complexities of flow profiles within a reactor. Initially CFD simulations only considered single-phase fluid systems, but the development of methodology and software has allowed for simulations of multiple fluid phases as well as multiphase fluid–solid simulations. Due to this development, CFD is an extremely useful simulation tool for particulate processing [31,32,33]. CFD simulations have been utilised in spherical agglomeration research to obtain the velocity profiles in the tank in order to calculate the size of bridging liquid droplets [11]. In the study by Orlewski et al., the calculated droplet size was much smaller than the final agglomerate size, suggesting that the agglomerates underwent the distribution mechanism and not the immersion mechanism [9,10].

To further investigate particulate processes, CFD simulations are often combined with other modelling techniques such as the Discrete Element Method (DEM) and PBM. Coupled CFD-PBM and CFD-DEM models have also been used for crystallisation and granulation processes in various equipment geometries, including stirred tanks, oscillatory baffled reactors and fluidized beds [31,34,35,36]. These processes are either a precursor to spherical agglomeration or mechanistically similar to spherical agglomeration and therefore are useful in understanding appropriate techniques for modelling spherical agglomeration. Whilst CFD-DEM simulations are extremely useful, they are very computationally expensive, limiting their potential applications to research.

A combined CFD-PBM using the Monte-Carlo method has been used for simulations of spherical agglomeration. The Monte-Carlo approach was used because it has low numerical load as it considers the particles as having individual properties, allowing a sample of 1000 particles to be representative of the full population, which could be much larger. This method was able to estimate how initial reagent distribution would influence the agglomeration; it also highlighted the importance of ensuring that the specified agglomeration kernel accurately reflects the agglomeration process, even if it increases the complexity of calculations [37].

As spherical agglomeration is a promising particle size enlargement technique that has been shown to improve micromeritic properties of different types of crystalline materials, it is important to investigate ways to optimise reactor design to improve agglomeration. Research into spherical agglomeration has focussed on determining the composition of the solvent system for various compounds including the bridging liquid-to-solid ratio, bridging liquid droplet size and bridging liquid injection position [1,2]. However, there has been little consistency in the equipment used for spherical agglomeration across studies. As spherical agglomeration occurs in a solvent system, the mixing behaviour in the tank is crucial to ensure good contact between the bridging liquid and primary crystals. However, there is little knowledge on how agitation influences particle position and fluid velocities in the tank as ensuring that the particles are well suspended and contact the bridging liquid is crucial for agglomerate formation. This work deploys both experimental and CFD analysis to increase understanding of how different impeller geometries, speeds and clearances influence mixing and particle velocity in stirred vessels and how this, in turn, influences the formation of spherical agglomerates. Increased understanding of the influence that stirred tank characteristics have on spherical agglomerate formation will allow for more informed reactor design choices for spherical agglomeration processes, which could increase the likelihood of scale-up of spherical agglomeration processes. Four impeller geometries were used at five different clearances and three impeller speeds. The 60 impeller configurations were tested for spherical agglomeration experiments with a model compound of polymethyl methacrylate (PMMA) beads in a water and toluene solvent system, with corresponding CFD simulations being performed in ANSYS Fluent [1].

## 2. Materials and Methods

### 2.1. Materials

The experiments were conducted as an agglomeration in suspension system, using monosized 52 μm diameter PMMA beads (Microbeads USA Spheromers CA 50, Lamberti SpA, Skedsmokorset, Norway), with a particle density of 1.2 g/cm^3^ and a bulk density of 0.6 g/cm^−3^, suspended in distilled water. Anhydrous 99.8% toluene (Sigma-Aldrich, St. Louis, MO, USA) was used as the bridging liquid as it has a contact angle of 8.7° with PMMA, which means it preferentially wets the PMMA particles, leading to agglomeration; the contact-angle measurement process and images can be seen in the Supplementary Information. Toluene has a density of 0.872 g/cm^3^ and a water solubility of 0.526 g/L at 25 °C. Toluene and water have an interfacial tension of approximately 35 mN/m [38]. As toluene is an ICH class II solvent, it is allowed to be used in pharmaceutical manufacturing; however, the concentration of toluene in the product must adhere to the ICH Q3C, USP <467> guidelines [39,40].

### 2.2. Experimental Methodology

For all experiments performed, 18 g of PMMA beads was suspended in 582 g of distilled water in a 1 L beaker with an overhead stirrer and four evenly spaced baffles to give a 3% wt/wt suspension. The beaker diameter was 90 mm, with a height of 144 mm. A solid loading of 3% wt/wt was chosen as previous studies have shown that this solid loading allows for successful agglomerate formation without increasing the agglomeration time [2,8]. The system was agitated for 10 min to allow it to equilibrate. After 10 min, 7.5 mL of toluene was added using a pipette to give instantaneous bridging liquid addition at a BSR of 0.5. BSR is the ratio of the bridging liquid volume to solids volume. The system was left under agitation for a further 45 min to allow for agglomerates to form, after which they were filtered using a Buchner funnel with a 90 mm diameter Millipore filter paper of 1.2 µm pore size and left to dry at 21 °C for 24 h. In the drying process of spherical agglomerates, the bridging liquid evaporates, but the liquid bridges between primary particles remain, resulting in robust spherical agglomerates.

To investigate the influence of impeller characteristics on spherical agglomerate formation, four impeller geometries were used at various impeller speeds and clearances. The four impeller geometries chosen were a flat-blade impeller, a propeller impeller, a Rushton turbine impeller, and a pitched-blade impeller. All impellers were stainless steel and had a blade diameter of 50 mm with a shaft diameter of 8 mm. These impellers were chosen as they promote different flow patterns in the system, with the flat-blade and Rushton turbine impellers promoting radial flow, whilst the propeller and pitched-blade impellers promote axial flow [20,41]. Each impeller geometry was tested at impeller speeds of 300 rpm, 450 rpm and 600 rpm and impeller clearances of 18 mm, 20 mm, 25 mm, 27 mm, and 30 mm to give impeller clearance/beaker diameter (C/D) ratios of 0.2 to 0.33; this is summarised in Table 1. For each impeller geometry, the experiment at an impeller speed of 450 rpm and clearance of 25 mm was performed in triplicate. It was chosen to perform the central conditions for impeller speed and clearance in triplicate for each impeller as in a central composite DoE, the consistency of the middle condition is repeated to determine an estimate for experimental error without needing to conduct repeats for all experiments [42]. For the repeats, the coefficient of variation for the repeated experiments was under 8% for all sieve samples, which suggests consistency of results, negating the need for other experimental repeats [43].

### 2.3. Product Characterisation

Once dry, the agglomerates were sieved using a Retsch AS 200 control sieve shaker (Retsch-Alle, Haan, Germany) at an amplitude of 50 Hz for 15 min. Due to the robust nature of the dried agglomerates, they can be transferred directly from the filter cake to the sieve shaker. The sieve sizes used were 150 µm, 250 µm, 355 µm, 425 µm, 500 µm, 600 µm, 710 µm, 850 µm, 1 mm, 1.18 mm, 1.4 mm, 1.7 mm and 2 mm. The mass retained on each sieve was taken and the calculations in Equations (3)–(7) were performed to give the mean particle size.

(3) $$m_{i}=m_{sp,i}-m_{s,i}$$

(4) $$y_{m,i}=\frac{m_{i}}{\sum m_{i}}$$

(5) $$f_{m,i}=\frac{y_{m,i}}{∆x_{i}}$$

(6) $$y_{i}=\frac{y_{m,i}{\bar{x_{i}}}^{-3}}{\sum y_{m,i}{\bar{x_{i}}}^{-3}}$$

(7) $$d_{43}=\frac{\sum \left(y_{m,i}{\bar{x}}_{i}\right)}{\sum y_{m,i}}$$

where $m_{i}$ is the mass of powder in size interval i, $m_{sp,i}$ is the mass of the sieve and powder recorded for sieve size i, $m_{s,i}$ is the mass of sieve size i, $y_{m,i}$ is the mass fraction of particle size i, $f_{m,i}$ is the mass frequency of particles of size i, $∆x_{i}$ is the difference between the interval of size i and i+1, $y_{i}$ is the number fraction in size interval i, ${\bar{x}}_{i}$ is the average of the interval i and i+1, and $d_{43}$ is the mean particle size of the distribution.

*d*_43_ was used in this study to determine whether the impeller was able to successfully produce agglomerates. If the value of *d*_43_ is within the size range of 500 μm to 1000 μm, then the impeller conditions promote spherical agglomeration. This consideration is due to the agglomerate diameter being 10–20 times larger than the diameter of the primary material, suggesting that sufficient growth has occurred without promoting overgrowth to form a few very large agglomerates. This size range also falls within the size range of 200–4000 µm that is commonly used for granules in the pharmaceutical industry [44,45,46].

Agglomerates were imaged using a Pixelink D775CU-T camera (Pixelink, Ottawa, ON, Canada) with a TV lens 1/3” CS, and the images were captured using UScope x64 software.

### 2.4. CFD Methodology

**Figure 1:**
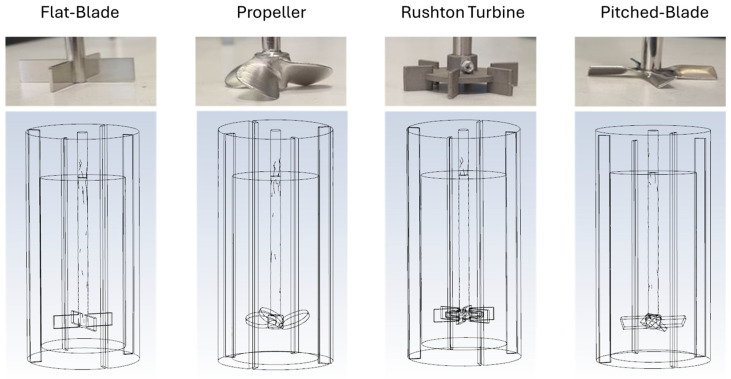
The four impeller geometries used in the investigation, and the corresponding CAD drawings that were produced for the CFD simulations based on the dimensions specified in Table 2.

ANSYS Fluent 19.1 was used for CFD simulations as it is a commercially available CFD package with integrated geometry construction and meshing. To produce the 60 corresponding CFD simulations to the experiments specified in Table 1, 20 reactor geometries were produced in ANSYS Design Modeller 2019 R2. The tank dimensions are shown in Table 2 and the CAD drawings for a 25 mm clearance for each impeller geometry are shown in Figure 1.

Agglomeration in suspension occurs in a multiphase system in which particles are suspended in a dispersion medium, and a bridging liquid is added to induce agglomeration. To accurately reflect this, the CFD simulations must consider multiphase flow. There are two main multiphase flow systems used in CFD simulations, namely Eulerian–Eulerian and Lagrangian. In the Eulerian–Eulerian multiphase model, the solid particles are treated as a continuous phase [47]. The different phases are represented by their volume fraction at different points in the system. The mass and momentum balances are solved for each of the phases in the system using a Reynolds-averaged approach [48]. The Lagrangian model considers solid material as individual particles and approximates hydrodynamic forces using single-particle empirical models [47]. Multiple studies have determined that a Lagrangian approach requires greater computational time than a Eulerian–Eulerian approach; therefore, a Eulerian–Eulerian multiphase model was selected for this work [49,50]. Equations (8) and (9) show the continuity and momentum equation respectively; these equations are based on the principles of conservation of mass and momentum.

(8) $$\frac{\partial p}{\partial t}+\rho \left(\nabla .\vec{v}\right)=0$$

(9) $$\rho \frac{\partial \vec{v}}{\partial t}=-\nabla p+\mu \nabla^{2}\vec{v}+\rho \vec{g}$$

where ρ is the density, $\vec{v}$ is the velocity, ∇ is the gradient operator and $\rho \vec{g}$ is the gravitational force per unit volume.

As spherical agglomeration occurs in a stirred tank, the system will be in turbulent flow. Various turbulence models were investigated to determine their applicability for this work. The Large-Eddy Simulation (LES) method was considered due to its high accuracy and versatility, especially for novel reactor configurations and extreme flow conditions [51]. However, this method is computationally expensive as it requires high mesh density to compute reliable results; it is also challenging to implement LES on unstructured meshes, with very fine meshes being required which would lead to a large increase in computation time [52]. Another turbulence model that is commonly used for CFD simulations of stirred tanks is the k-ε turbulence model. This model assumes that the average velocity gradient is proportional to the Reynolds stress [53]. In this model, the turbulent kinetic energy averages the fluctuating turbulent velocity in three directions. The turbulent dissipation is represented by ε [54]. The k- ε turbulence model is popular due to it having good accuracy with a lower computation time than other turbulence models [53]. The k-ε turbulence model has produced some inaccurate results for recirculating flows, leading to variations of the k-ε model being developed [55]. The LES method was not considered appropriate for this work as it would require a very fine mesh, greatly increasing the computational cost of the simulations. The k-*ε* turbulence model was chosen for this work as it is a well-established method for engineering flow processes with relatively low computation expense compared to other turbulence models [55,56].

To increase the accuracy of the k-ε turbulence model, the mesh near the wall needs to be refined. Therefore, a mesh density analysis was performed; it determined that a 4 mm tetrahedral mesh with contact sizing, patch conforming and inflation around the tank walls and impeller blades produced accurate results (details of the mesh density analysis are provided in the SI). The rotation of the impeller was simulated using frame motion of a cylindrical zone in the reactor. CFD simulations were validated by recording stirred suspensions of similarly sized beads in water and tracking specific tracer beads to determine whether the CFD velocity and suspension profiles were the same; details of this can be seen in the Supplementary Information, with more detail in [1].

The CFD simulations investigated the suspension behaviour of monosized 500 µm diameter spherical particles of PMMA in water with different impeller geometries, clearances and speeds. These conditions were chosen to investigate the suspension behaviour of agglomerates once they are formed. Virtual mass modelling was used to account for the phase interactions of the particles and the water with a no-slip boundary condition on the tank walls.

## 3. Results

### 3.1. Experimental Results

**Figure 2:**
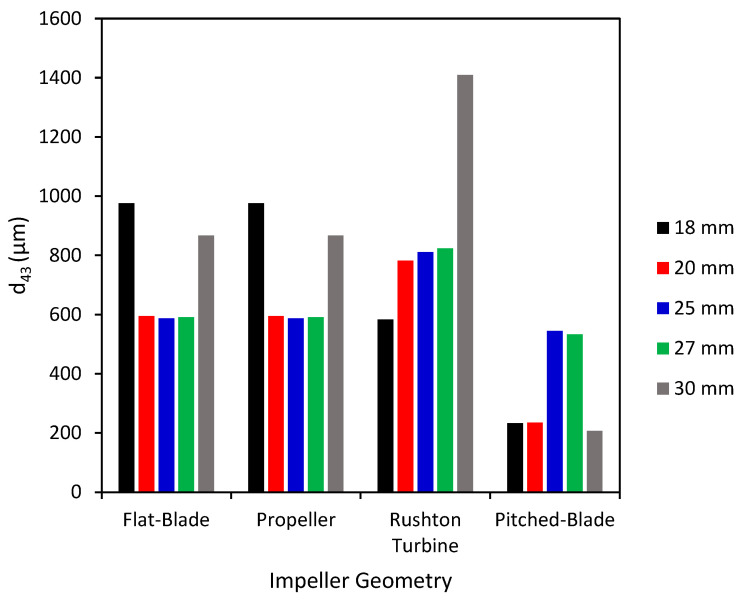
Average particle size (*d*_43_) for agglomerates produced at 300 rpm with different impeller geometries and clearances.

**Figure 3:**
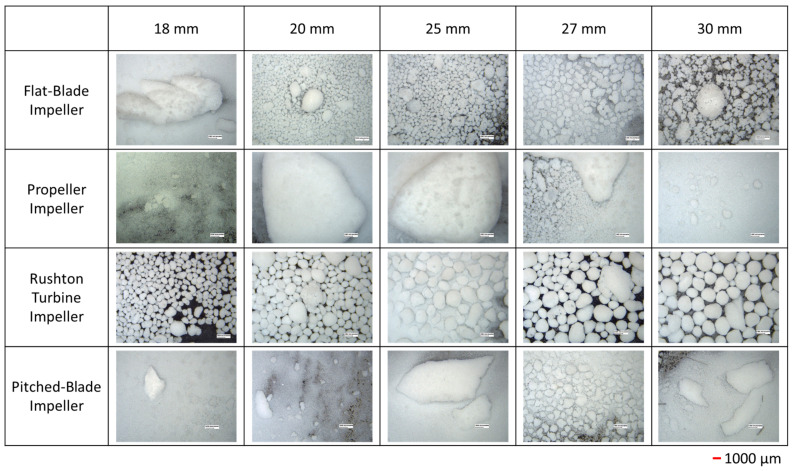
Images of agglomerates produced at 300 rpm for different impeller geometries and clearances. Each image is the equivalent of 1.13 cm high and 1.49 cm wide.

The *d*_43_ of agglomerates produced with the four impeller geometries at an impeller speed of 300 rpm and varied clearances are shown in Figure 2. The PSD graphs for all impeller geometries, speeds and clearances can be seen in the SI. Agglomerates produced by the flat-blade impeller at 300 rpm and different clearances consistently have *d*_43_ between the desired range of 500–1000 µm, with the highest value of *d*_43_ being at a clearance of 18 mm for the flat-blade impeller. From Figure 3 it can be seen that although the value of *d*_43_ lies within the range, the agglomerates formed are neither consistent in size nor spherical, with there being a few very large agglomerates and a lot of fine powder. The results also show that the *d*_43_ of particles produced with the propeller impeller are inconsistent across the various impeller clearances. The agglomerate images in Figure 3 show that the propeller impeller produces a few very large agglomerates whilst a large amount of the primary material remains in the system. The inconsistent size and shape of agglomerates produced by the propeller impeller may be caused by this impeller imparting low levels of shear into the system that are insufficient to induce consolidation of the agglomerates [57].

From Figure 2, the Rushton turbine impeller produces agglomerates with a *d*_43_ that is fairly consistent for clearances of 20 mm to 27 mm. There is a large increase in *d*_43_ when the impeller clearance is increased to 30 mm; this is at a C/D ratio of 0.33. It has been observed in previous studies that a Rushton turbine impeller positioned at a C/D < 0.3 does not allow for double-loop formation [27,28]. Of the four impeller geometries tested, the pitched-blade impeller has the lowest *d*_43_. The agglomerate images in Figure 3 show that an impeller speed of 300 rpm is too low to successfully produce agglomerates with consistent size and sphericity, with the Rushton turbine impeller producing agglomerates that are more consistent in size and sphericity than those produced using the other impellers.

**Figure 4:**
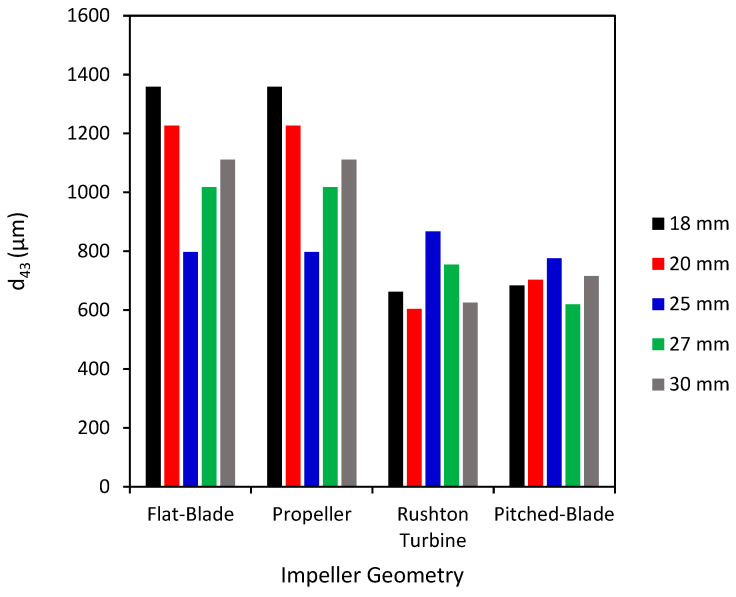
Average particle size (*d*_43_) for agglomerates produced at 450 rpm with different impeller geometries and clearances.

Figure 4 shows the *d*_43_ of agglomerates produced using the various impeller geometries and clearances at a speed of 450 rpm. At an impeller speed of 450 rpm, the *d*_43_ of the agglomerates produced by the pitched-blade impeller is increased when compared to the agglomerates at 300 rpm (Figure 2). The propeller impeller has fluctuations in the values of *d*_43_ at varied impeller clearance values, suggesting inefficient mixing between the particles and bridging liquid.

**Figure 5:**
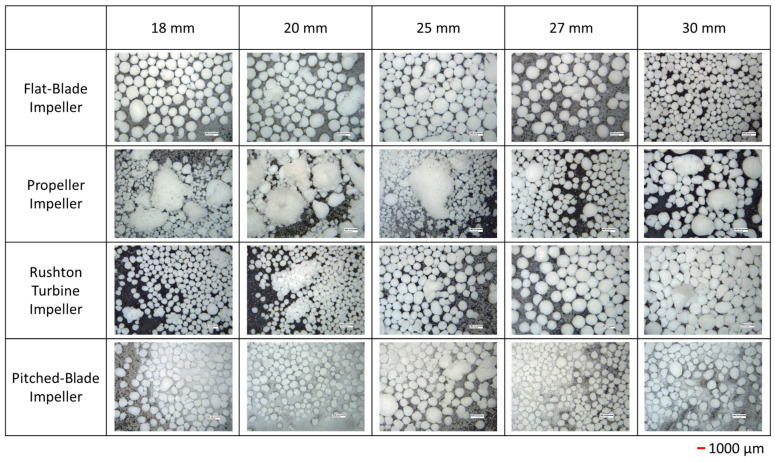
Images of agglomerates produced at 450 rpm for different impeller geometries and clearances. Each image is the equivalent of 1.13 cm high and 1.49 cm wide.

Values of *d*_43_ for the flat-blade impeller are consistently higher with an impeller speed of 450 rpm compared to 300 rpm for all impeller clearances. This can be seen when comparing the agglomerate images at 300 rpm (Figure 3) to the images at 450 rpm (Figure 5), as at the lower impeller speed, there was still a lot of fine powder in the agglomerate images with a few very large agglomerates produced at 300 rpm. In Figure 5 it can be seen that there is a lot less fine powder in the images but that there are more agglomerates formed. Although these are not consistent in size, there will be a much narrower particle size distribution than the agglomerates produced at 300 rpm.

From the agglomerate images in Figure 5, an impeller speed of 450 rpm produces agglomerates of consistent particle size and increased sphericity for all four impeller geometries when compared to the agglomerates formed at 300 rpm (Figure 3). The agglomerates produced by the propeller impeller at 450 rpm appear less consistent in their size and sphericity than agglomerates produced by the other impellers. However, increasing the impeller speed appears to reduce the number of fines produced using the propeller impeller as the values of *d*_43_ in Figure 4 are higher than those in Figure 2 for all clearances except 25 mm. The higher impeller speed results in increased values of particle collision velocity, which increases the adhesive forces between agglomerates [12,58]. The agglomerates produced using the pitched-blade impeller at 450 rpm have an average particle size between 600 µm and 800 µm for all impeller clearances, with the clearance of 27 mm producing the smallest average agglomerate size. From Figure 5, the agglomerates produced at a clearance of 27 mm and a speed of 450 rpm have increased sphericity in comparison with agglomerates produced at different clearances with a speed of 450 rpm.

**Figure 6:**
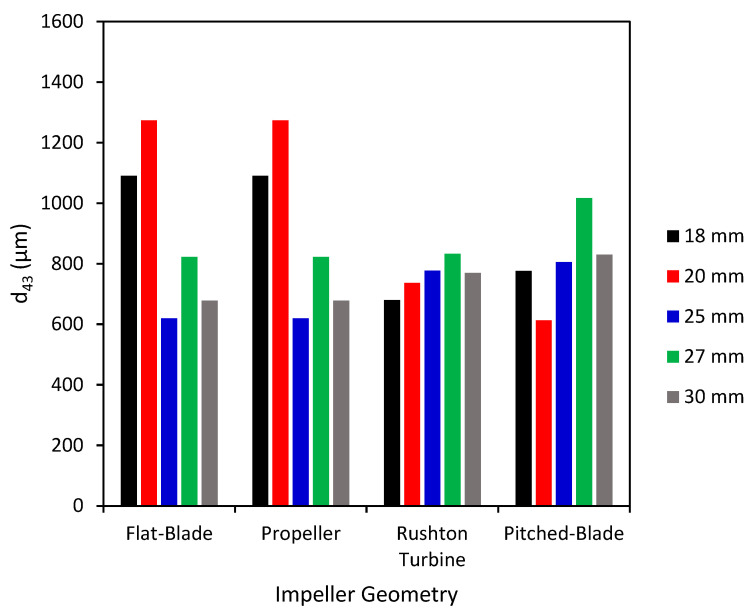
Average particle size (*d*_43_) for agglomerates produced at 600 rpm with different impeller geometries and clearances.

**Figure 7:**
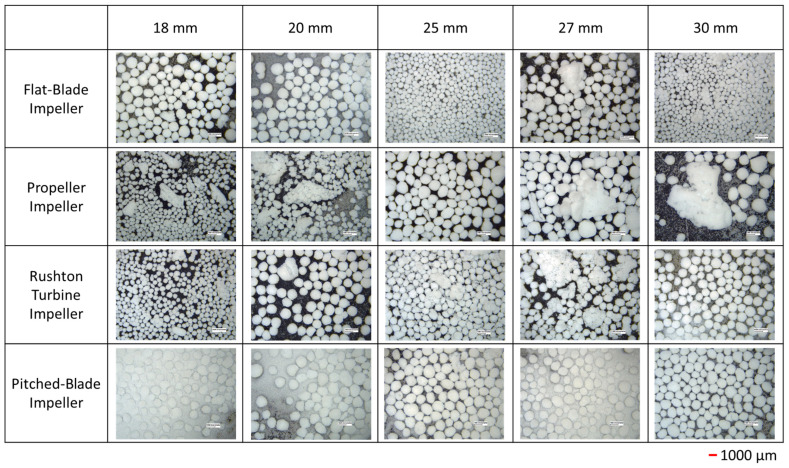
Images of agglomerates produced at 600 rpm for different impeller geometries and clearances. Each image is the equivalent of 1.13 cm high and 1.49 cm wide.

Figure 6 shows the average particle size of the agglomerates produced at 600 rpm. The agglomerates produced by a flat-blade impeller at 600 rpm have a smaller *d*_43_ than agglomerates produced at 450 rpm (Figure 4). This may be due to the increased impeller speed resulting in greater shear forces in the system. The shearing effects will promote agglomerate consolidation and could lead to breakage [21]. As agglomerate breakage has not been studied, it is also possible that the shear induced by increased impeller speed may result in breakage of the bridging liquid droplets. Smaller droplets of bridging liquid would lead to smaller agglomerates [11]. As Figure 7 shows, the agglomerates produced by the Rushton turbine at 600 rpm are consistently spherical. Rushton turbine impellers have high power numbers and, therefore, increased power consumption [20]. It has also been found that increased Rushton turbine clearances increase the power requirements [24]. In the spherical agglomeration PBM developed by Ahmed et al., the power consumption is used to estimate the energy dissipation in the system [12]. Energy dissipation is used for calculating the velocity of particle and fluid interactions, as well as the separation force [10,12,58]. Therefore, increased clearance will increase the power consumption in the system, resulting in higher velocities in the system. Increased velocities will result in more successful collisions and consolidation of the agglomerates.

**Figure 8:**
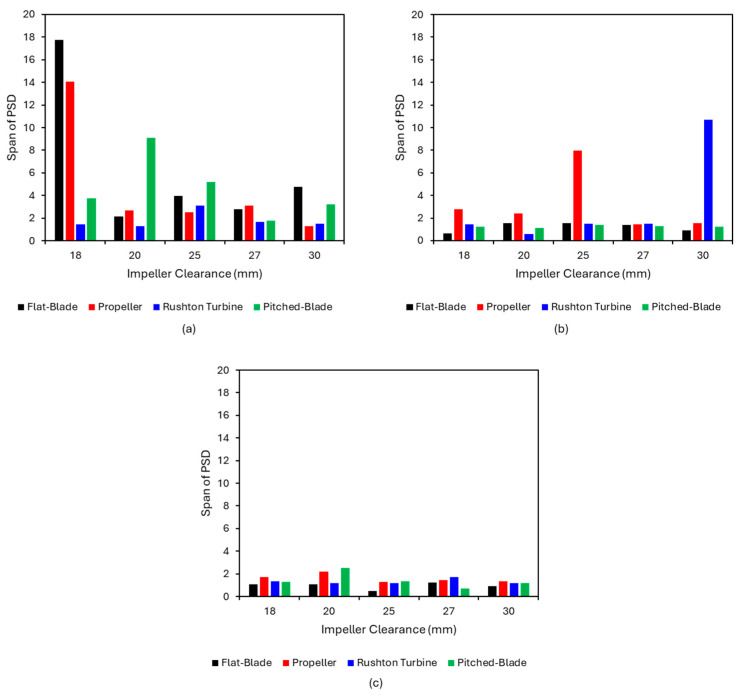
Span of the particle size distribution for the four impeller geometries at different clearances and impeller speeds: (**a**) 300 rpm, (**b**) 450 rpm and (**c**) 600 rpm.

Equation (10) is used to calculate the span of the PSD; the results of this are shown in Figure 8.

(10) $$Span=\frac{d_{90}-d_{10}}{d_{50}}$$

where $d_{90}$ is the diameter that 90% of the distribution is smaller than (μm), $d_{50}$ is the median of the particle size distribution (50% of the particles are smaller than this size (μm)), and $d_{10}$ is the diameter that 10% of the particles are smaller than (μm).

Figure 8 demonstrates a clear link between the impeller speed and agglomerate size consistency. At an impeller speed of 300 rpm (Figure 8a), there is more variation in PSD for the different impeller geometries and clearances than at higher impeller speeds, suggesting that an impeller speed of 300 rpm is not able to successfully produce consistent spherical agglomerates. The increased impeller speeds producing more consistent agglomerates could be due to increased speeds improving the suspension of particles [59]. At increased impeller speeds, the impeller tip speed is higher, resulting in higher shear forces in the system. Increased shear forces exponentially increase the agglomeration rate constant, resulting in faster agglomeration, leading to increased contact between the bridging liquid and particles [23]. Increased impeller speeds also increase agglomerate consolidation, resulting in increased sphericity [21]. However, shear can result in more breakage, leading to a broader particle size distribution [21,22].

In Figure 8 the span of the particle size distribution for the Rushton turbine impeller at a clearance of 30 mm shows interesting behaviour. As the impeller speed increases from 300 rpm to 450 rpm, the span increases from approximately 2 to 10; at an increased impeller speed of 600 rpm, the span has reduced to 1. It is likely that as the impeller speed increases from 300 rpm to 450 rpm, the shear forces increase, resulting in increased agglomerate breakage and a wider PSD. As the impeller speed is further increased to 600 rpm, the increased shear forces result in faster agglomeration and increased consolidation, resulting in smaller, more consistently sized particles; this can be seen in the agglomerate images in Figure 3, Figure 5 and Figure 7.

### 3.2. CFD Results

The experimental results in Section 3.1 demonstrated that the mixing profile in the tank is extremely important for successful spherical agglomeration. CFD simulations were performed that corresponded to the stirred tank geometries that were used in the experiments to be able to analyse the velocity profile and particle suspension in the tank.

An important parameter in determining the extent of particle suspension is the just-suspended impeller speed, defined in Equation (2). This parameter is an indicator of the minimum impeller speed required for there to be no particles settled at the bottom of the tank for over 2 s. The results of Equation (2) can be used to calculate the velocities in the tank that would be sufficient for particle suspension using Equation (11).

(11) $$I_{js}=d\pi \frac{N_{js}}{60}$$

where $I_{js}$ is the just-suspended impeller tip speed (m/s), d is the impeller diameter (m), and $N_{js}$ is the just-suspended impeller speed (rpm) calculated using Equation (2).

**Figure 9:**
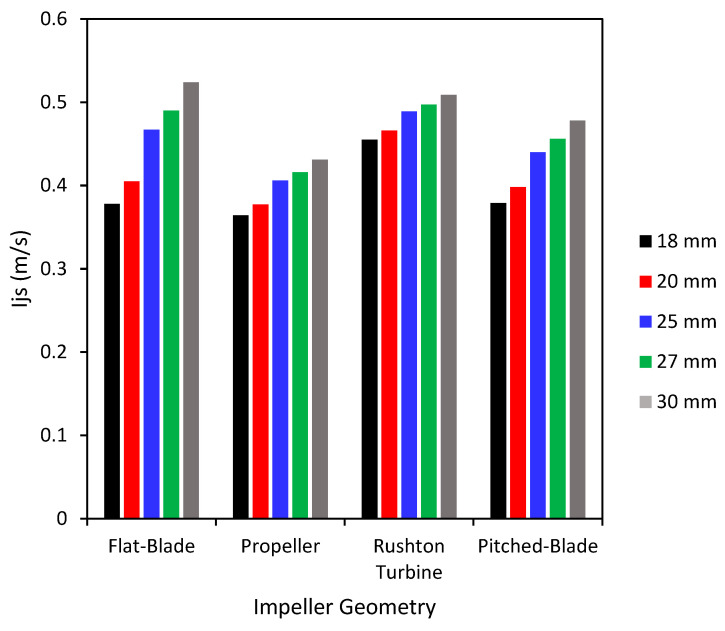
Calculated values of just-suspended impeller tip speed for the impellers at different clearances.

The results of Equation (11) are shown in Figure 9, where it can be seen that the just-suspended impeller tip speed increases with impeller clearance as the Zwietering constant (S in Equation (2)—calculation of $N_{js}$) is dependent on impeller geometry, liquid level, impeller diameter and impeller clearance [25]. The three impeller speeds tested were 300 rpm, 450 rpm and 600 rpm; these values have a calculated impeller tip speed of 0.79, 1.18 and 1.57 m/s respectively, which is greater than the values of $N_{js}$ that were calculated using Equation (2). With an impeller speed above 450 rpm, the tip speed is over double the needed just-suspended impeller speed, suggesting that all particles will be well suspended and able to mix with the bridging liquid. This suggests that the particles in the spherical agglomeration experiments will be well suspended, leading to increased contact between particles and bridging liquid, increasing the likelihood of successful spherical agglomerate production. The full results for the calculation of $N_{js}$ and $I_{js}$ can be seen in the Supplementary Information.

From the CFD simulations, the volume-weighted average (VWA) velocity magnitude for the stirred tank was obtained for all impeller geometries, speeds and clearances tested. The VWA was used to analyse the CFD results because it considers the size of individual cells as it calculates the average of the specified cell zone, in this case the stirred tank. This means that the larger cells in the tank will hold more weight than smaller cells, resulting in increased accuracy of the result.

**Figure 10:**
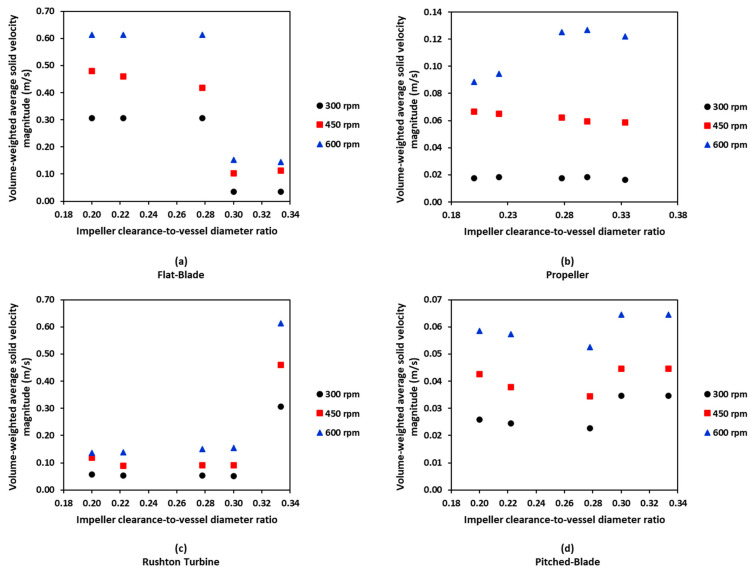
VWA solid velocity magnitude for the four impeller geometries tested at different impeller speeds and clearances.

Figure 10 shows that the four impeller geometries produce different values of VWA solid velocity magnitude at all impeller speeds and clearances. For all impeller geometries, increased impeller speed results in higher values of VWA solid velocity magnitude. It is expected that higher impeller speeds would result in increased VWA velocity magnitude as the impeller will be rotating at higher tip speeds, resulting in increased velocities in the rest of the tank.

**Figure 11:**
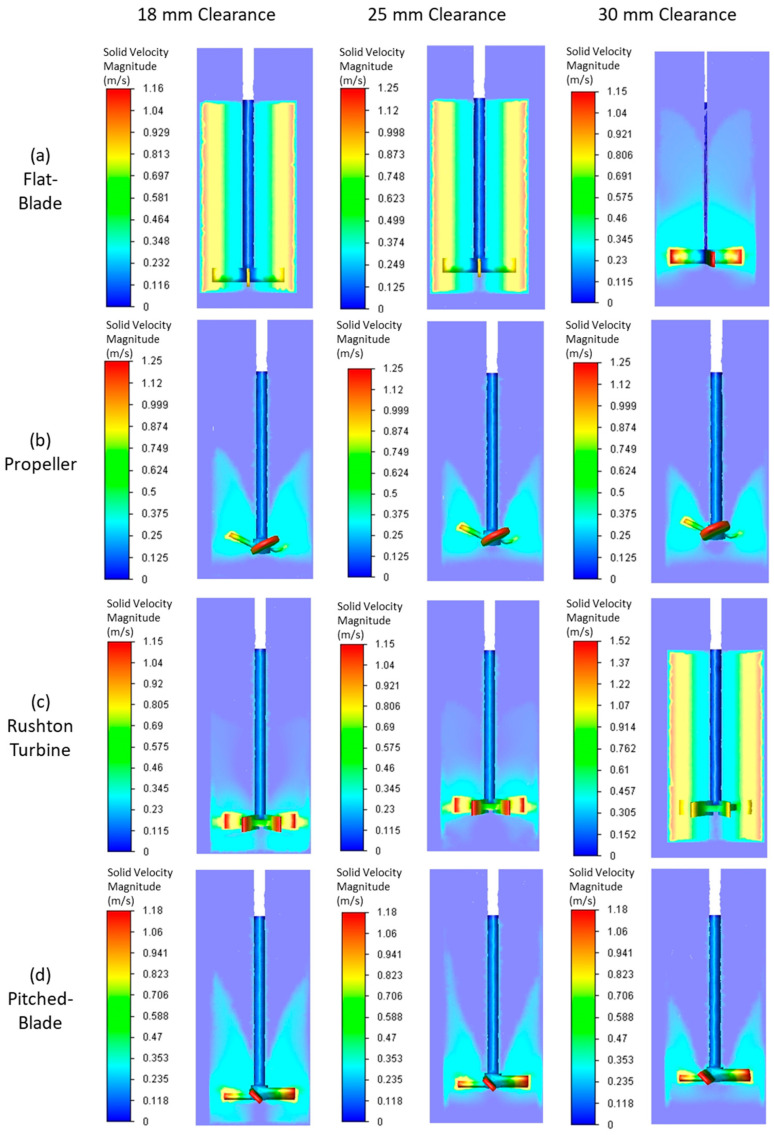
CFD contours of the velocity magnitude of the particles in the tank for the four impeller geometries at various clearances at an impeller speed of 450 rpm.

The flat-blade impeller VWA solid velocity magnitude decreases with an increase in C/D ratio for the three impeller speeds tested. At 300 rpm, the VWA solid velocity magnitude for a flat-blade impeller is lower than the calculated $I_{js}$ value for all clearances. This suggests that particles at this impeller speed will not be well suspended in the tank, resulting in poor agglomeration. This is consistent with experimental results (Figure 3), showing that agglomerates produced using a flat-blade impeller at 300 rpm were inconsistent in both size and sphericity. As Figure 10a shows, for all impeller speeds tested with the flat-blade impeller, the VWA velocity magnitudes at low C/D values are higher than, or close to, the $I_{js}$ values for clearances between 18 and 30 mm. The CFD contours for the flat-blade impeller at 450 rpm are shown in Figure 11a. In the contours, there is a clear difference in the solid velocity magnitude profile at different C/D ratios. The lower C/D ratios have a more uniform flow distribution with areas of high velocity. For a C/D of 0.33, the flow pattern is closer to the impeller and there are lower particle velocities at increased clearances. It was also found that the VWA velocity magnitude for both the solid and the liquid decreases at C/D > 0.3. In a study by Devarajulu & Loganathan, it was observed that a flat-blade impeller with six blades was effective at suspending solids at C/D < 0.25, suggesting that lower clearances would favour a flat-blade impeller [25].

A propeller impeller promotes axial flow in the tank. For the propeller impeller, the solid particle VWA velocity magnitude increases with increased impeller speed for all values of C/D. This is expected as increased impeller speed will lead to a higher impeller tip speed, resulting in greater velocity in the system [23]. As Figure 10b shows, for all impeller speeds and clearances, VWA solid velocity magnitudes for a propeller impeller are lower than the calculated value of $I_{js}$ that can be seen in Figure 9. At higher clearances, the VWA solid velocity magnitude is much lower than the $I_{js}$ values, suggesting that more particles have settled to the bottom of the tank. At an impeller speed of 600 rpm, the VWA solid velocity magnitude increases with impeller C/D values. This suggests that agglomerate formation in the system will improve with an increased clearance at 600 rpm. The increase in particle suspension at C/D > 0.28 may be due to a trend observed by Kresta and Wood in 1993 [60]. In this work, they determined that for a pitched-blade impeller, when C/D > 0.3, the single-loop flow pattern reaches the top and bottom of the tank. As a pitched-blade impeller also imparts axial flow in a stirred tank, it can be assumed that C/D will have a similar influence for flow generated by a propeller impeller [60]. Figure 11b shows solid velocity magnitude CFD contours for the propeller impeller at a speed of 450 rpm. The shape of the flow profile is fairly consistent across C/D ratios for an impeller speed of 450 rpm, and it does not cover a large area within the tank. This suggests that a propeller impeller is not an effective choice for spherical agglomeration at the tested impeller speeds of 300–600 rpm as it produces velocities well below the critical impeller tip speed, resulting in inconsistent mixing between particles and bridging liquid. Increased impeller speeds may lead to increased agglomerate consistency when using a propeller impeller.

The Rushton turbine impeller promotes radial flow in the system [20]. The VWA solid velocity magnitude profile for a Rushton turbine at different impeller speeds and clearances is shown in Figure 11c. It can be seen that for all impeller speeds, the highest value of VWA solid velocity magnitude for a Rushton turbine is at a C/D of 0.33. This value is much greater than values at lower C/D values. The increase in VWA solid velocity magnitude correlates to studies by Montante et al. and Zhu et al. [27,28]. In this research and the studies by Montante et al. and Zhu et al., it was found that C/D for a Rushton turbine has a large influence on the flow pattern in the tank. At C/D values below 0.3, the system operates with a single-loop flow pattern, similar to that induced by an axial flow impeller [27,28]. Increasing the clearance to give C/D > 0.3 will lead to the formation of the double-loop flow pattern that is expected from a radial impeller. The flow pattern formed using a Rushton turbine impeller at various C/D ratios and an impeller speed of 450 rpm is shown in Figure 11c. A C/D of 0.33 produces a flow profile that is very different to the profiles at lower C/D values for all impeller speeds. This is due to the trend also observed by Montante et al., 1999 and Zhu et al., 2019, in which a C/D > 0.3 allows for a double-loop flow pattern to form when a Rushton turbine is used; at lower clearances, the Rushton turbine will produce a single-loop flow pattern, similar to that of an axial impeller [27,28]. The results obtained at a C/D of 0.33 have a higher maximum solid velocity magnitude than the lower clearances, suggesting that the double-loop pattern induced at this clearance will result in higher velocities in the system.

A pitched-blade impeller promotes axial flow in the stirred tank. The VWA solid velocity magnitude for a pitched-blade impeller at different impeller speeds and C/D ratios can be seen in 10d. The VWA solid velocity magnitude for all impeller speeds decreases with an increase in clearance up to C/D < 0.3. After this, the VWA solid velocity magnitude increases. Increased clearances for a pitched-blade impeller have been shown to increase the impeller power number [25]. The spherical agglomeration PBM developed by Ahmed et al. uses power consumption in the calculation of the velocity of solid and liquid interaction, as well as separation forces [12]. In the model, increasing power consumption would increase the velocities in the system. This suggests that spherical agglomerate production with a pitched-blade impeller will be improved at increased C/D values as the velocities in the system will be greater. Figure 11d shows the CFD contours of a pitched-blade impeller operating at various C/D ratios and an impeller speed of 450 rpm; the flow pattern appears to cover less area in the tank as C/D increases. This may be due to the increase in C/D leading to particles that have settled towards the bottom of the tank not becoming entrained in the flow pattern and, therefore, not becoming suspended. Increased C/D for a pitched-blade impeller has been shown to extend the height of the flow pattern, with a C/D > 0.3 covering the full liquid height [60]. The flow pattern for a C/D of 0.33 at an impeller speed of 450 rpm does not reach this height, suggesting that the velocity imparted on the particles is too low for them to stay suspended as they move further from the impeller.

## 4. Discussion

From the CFD results for the four impeller geometries tested, it was found that as the impeller speed is increased, the VWA solid velocity magnitude also increases. These results are to be expected as the increased impeller speed will increase the impeller tip speed, which will result in faster velocities in the tank. It was determined in the experimental study that an impeller speed of 300 rpm was insufficient to produce spherical agglomerates for all impellers. However, the images in Figure 3 show better agglomerate formation with the Rushton turbine at 300 rpm when compared to the other three impeller types. The agglomerates formed at 300 rpm with the Rushton turbine also had the lowest values of particle size span, as can be seen in Figure 8a.

The flat-blade impeller had a transition of flow pattern when C/D > 0.3. This was observed in the VWA velocity magnitude graphs and the CFD contours in Figure 10a and Figure 11a, respectively. From the experimental results, this trend is harder to observe. The experimental images at 450 rpm and 600 rpm all show well-formed agglomerates that had consistent sphericity. This may be due to the VWA solid velocity magnitude for a flat-blade impeller being greater than the values for the propeller and pitched-blade impellers at all impeller speeds; this can be seen in Figure 10. As the system is operating at a greater velocity than the axial flow impellers, it can produce agglomerates that are spherical in shape and fairly consistent in size.

**Figure 12:**
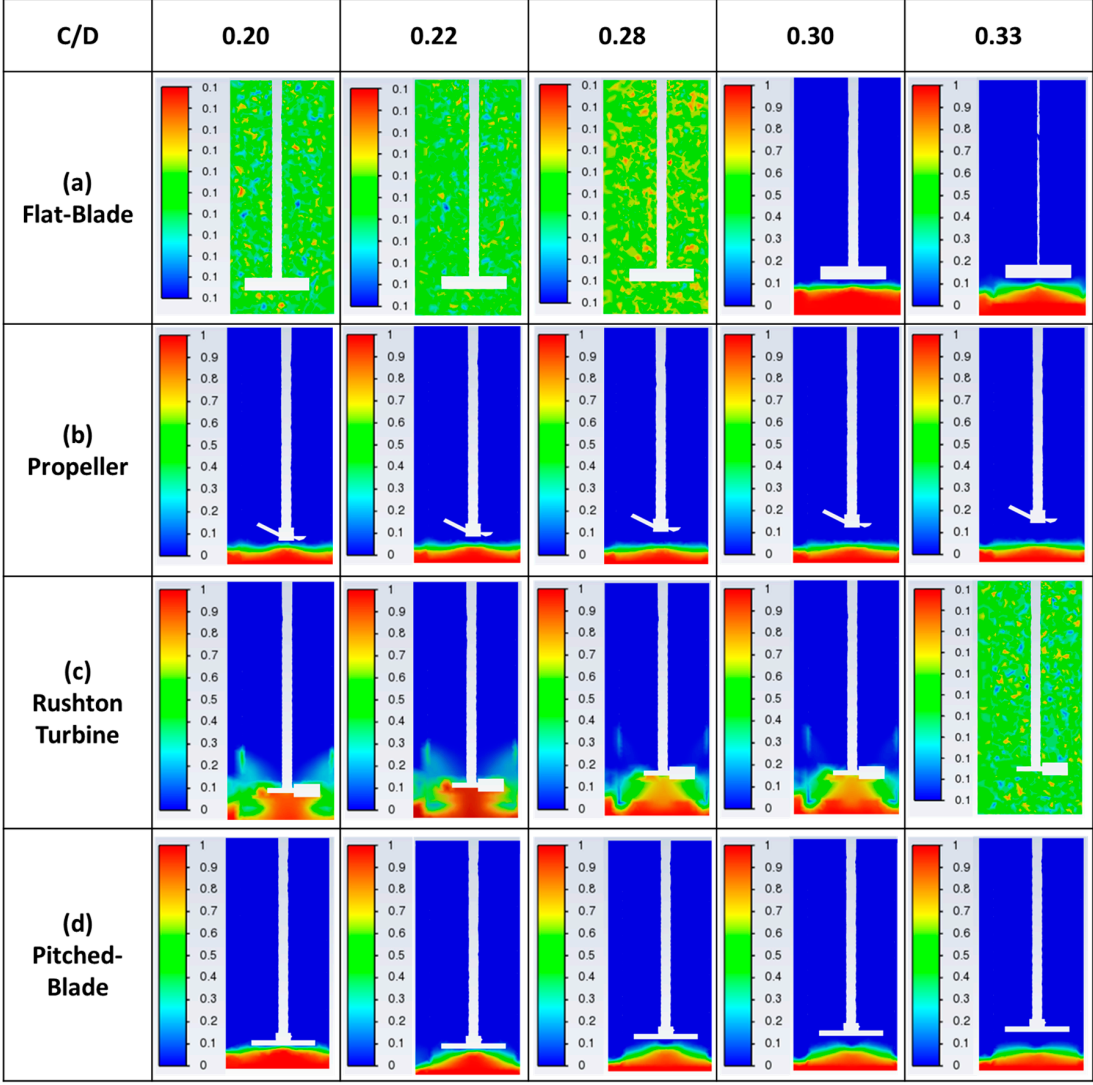
CFD contours of the volume fraction of the solid particles in the tank for the four different impeller geometries at various clearances and an impeller speed of 450 rpm.

In the experimental study, the propeller impeller produced very large agglomerates, whilst still having a large portion of primary material left in the product. The flow pattern of the four impeller geometries investigated in this work were studied by Matzke et al. in 2022, this study found that even at high Reynolds numbers, the propeller impeller had the shortest circulation loop of the four impellers [61]. The short circulation loop would result in poor contact between the bridging liquid and particles, resulting in limited spherical agglomeration for the propeller impeller. Figure 11 and Figure 12 show that the movement of particles in the tank is limited with a propeller impeller, suggesting that there are more dead zones in the reactor, leading to a few very large agglomerates and substantial unagglomerated primary material in the tank.

The pitched-blade impeller was not consistent at producing spherical agglomerates in the experimental study. From the CFD simulations, it appeared that high C/D values and increased impeller speeds were the most effective conditions for spherical agglomerate formation. The experimental results for the pitched-blade impeller were most consistent for impeller speeds of 450 rpm and 600 rpm. Of the pitched-blade experiments, the ideal configuration based on the agglomerate images and the value of *d*_43_ was determined to be at a C/D of 0.33 with an impeller speed of 600 rpm. In the CFD study, this condition produced the greatest VWA velocity for both the solid and the liquid, as well as the flow pattern that covered the most area of the fluid in the tank for this impeller.

In the experimental study, the Rushton turbine was the best-performing impeller for spherical agglomerate production. The CFD contours in Figure 11c show that for C/D < 0.3, the flow pattern is similar to that of an axial impeller, which is thought to be more effective at particle suspension than the conventional double-loop pattern produced by a radial impeller [27,28]. The contours for the Rushton turbine show that the solid velocity magnitude is higher both above and below the impeller, demonstrating that particles that were settling towards the bottom of the reactor will be caught in this flow at the bottom of the tank. This will result in them becoming entrained in the flow field and, therefore, undergoing agglomeration. For the Rushton turbine at a C/D of 0.33, the flow pattern is different. This flow pattern, shown in Figure 11c, does not reach the bottom of the tank. However, it does cover a substantial portion below the impeller, and it reaches the full height of the liquid in the tank. The velocities for this C/D ratio are also higher than for lower C/D values at the same speed. This suggests that whilst the flow may not reach the bottom of the tank, particles are unable to settle, especially at 600 rpm, which has a VWA solid and liquid velocity magnitude greater than the calculated value of suspension velocity ($I_{js}$), as shown in Figure 9.

## 5. Conclusions

The impeller geometry has an influence on the formation of spherical agglomerates, with the experimental results suggesting a clear correlation between increased impeller power number and the consistency of agglomerate shape and size. From the CFD study, the velocities in the tank are greater for radial flow-promoting impellers than axial flow-promoting impellers. At 600 rpm, the velocity magnitude for the pitched-blade and propeller impellers are below 0.1 m/s for all impeller clearances. The velocity magnitudes for the Rushton turbine and flat-blade impellers (0.15 to 0.65 m/s) are consistently higher than the axial flow impellers, even when the flow regime for the radial flow impellers transitions to axial flow. The increased velocities will result in increased collision velocity between particles, resulting in a greater number of successful collisions, leading to improved spherical agglomerate consistency. The flat-blade impeller and Rushton turbine impeller have lower span values than the axial impellers at impeller speeds greater than 450 rpm, due to the formed agglomerates being more consistent in size. These agglomerates are also consistently spherical in shape.

This work demonstrates the strong influence of mixing characteristics in the stirred tank on agglomerate formation, and impeller design, position and speed must be carefully considered when designing a spherical agglomeration process. In this work, impellers that promote radial flow were shown to produce agglomerates that were more consistent in size and sphericity than axial flow-promoting impellers, suggesting that radial flow impellers should be used for spherical agglomeration. As with the impeller geometry, clearance and speed have such a large influence on the agglomerate characteristics that they need to be included in models for spherical agglomeration. Existing models can be further developed by incorporating the impeller geometry and clearance, utilising the findings from this work.

## Figures and Tables

**Table 1:** Summary of experiments and CFD simulations performed. Each impeller speed and clearance value were tested for all four impeller geometries. (*) denotes experiments performed in triplicate.

Impeller Geometry	Number of Blades	Blade Pitch (°)	Published Impeller Power Number	Impeller Speed (rpm)	Impeller Clearance (mm)				
Flat-Blade	4	90	3 [20]	300	18	20	25	27	30
				450	18	20	25 *	27	30
				600	18	20	25	27	30
Propeller	3	30	1–1.1 [30]	300	18	20	25	27	30
				450	18	20	25 *	27	30
				600	18	20	25	27	30
Rushton Turbine	6	90	4–5 [20,30]	300	18	20	25	27	30
				450	18	20	25 *	27	30
				600	18	20	25	27	30
Pitched-Blade	4	45	1.5 [20]	300	18	20	25	27	30
				450	18	20	25 *	27	30
				600	18	20	25	27	30

**Table 2:** Dimensions of the stirred tank that was used for the CAD drawings produced for the CFD simulations.

Description	Value (mm)
Vessel Diameter	90
Liquid Height	144
Vessel Height	180
Width of Impeller Blade	10
Impeller Diameter	50
Clearance	18, 20, 25, 27, 30
Baffle Width	9
Number of Baffles	4

## Data Availability

The original contributions presented in this study are included in the article and Supplementary Materials. The datasets used and/or analysed during the current study are available from the corresponding author upon request.

## Supplementary Materials

The following supporting information can be downloaded at: https://www.mdpi.com/article/10.3390/pharmaceutics18030300/s1, Figure S1: Contact angle of toluene on PMMA beads; Table S1: Contact angle measurements for toluene on the PMMA beads; Figure S2: The number of iterations for a simulation to converge to a result against the number of nodes in the mesh; Figure S3: The volume-weighted average velocity magnitude against the specified mesh size; Figure S4: A sequence of frames from the video used for CFD validation; Figure S5: PSD for agglomerates produced by the flat blade impeller at a speed of 300 rpm for different clearances; Figure S6: PSD for agglomerates produced by the flat blade impeller at a speed of 450 rpm for different clearances; Figure S7: PSD for agglomerates produced by the flat blade impeller at a speed of 600 rpm for different clearances; Figure S8: PSD for the propeller impeller with an impeller speed of 300 rpm at various clearances; Figure S9: PSD for the propeller impeller with an impeller speed of 450 rpm at varied impeller clearances; Figure S10: PSD for the propeller impeller at different clearances with an impeller speed of 600 rpm; Figure S11: PSD for the agglomerates produced with the Rushton turbine at different clearances with an impeller speed of 300 rpm; Figure S12: PSD for spherical agglomerates formed using a Rushton turbine at 450 rpm impeller speed, with various clearances; Figure S13: PSD of spherical agglomerates produced using a Rushton turbine at 600 rpm with various clearances; Figure S14: PSD for the spherical agglomerates formed with a pitched blade impeller at an impeller speed of 300 rpm at various impeller clearances; Figure S15: PSD for the pitched-blade impeller spherical agglomerates formed at impeller speed of 450 rpm and various impeller clearances; Figure S16: PSD of spherical agglomerates formed with a pitched blade impeller at different impeller clearances, with an impeller speed of 600 rpm; Table S2: Calculated values of just-suspended impeller speed and just-suspended impeller tip speed for the different impeller geometries and clearances.

## Abbreviations

The following abbreviations are used in this manuscript:

API	Active Pharmaceutical Ingredient
BSR	Bridging Liquid-to-Solid Ratio
C/D	Impeller Clearance-to-Tank Diameter Ratio
CFD	Computational Fluid Dynamics
CSTRs	Continuous Stirred Tank Reactors
LES	Large-Eddy Simulation
PBM	Population Balance Model
PMMA	Polymethyl Methacrylate
PSD	Particle Size Distribution
VWA	Volume-Weighted Average
