# Supplementary material for: A CFD and Experimental Investigation of the Influence of Flow Characteristics on Spherical Agglomeration
Source: Pharmaceutics. 2026 Feb 27;18(3):301. doi: 10.3390/pharmaceutics18030301 (PMC13029191; doi:10.3390/pharmaceutics18030301)
Supplement: Supplementary file 1 [file pharmaceutics-18-00301-s001.zip › Table S1.pdf]

Table S1 Contact angle measurements for toluene on the PMMA beads

	**52 µm PMMA Bead Contact Angle (°)**
Reading 1	8.67
Reading 2	8.35
Reading 3	8.96
Average	8.66
Standard Deviation	0.249
