# Supplementary material for: A CFD and Experimental Investigation of the Influence of Flow Characteristics on Spherical Agglomeration
Source: Pharmaceutics. 2026 Feb 27;18(3):301. doi: 10.3390/pharmaceutics18030301 (PMC13029191; doi:10.3390/pharmaceutics18030301)
Supplement: Supplementary file 1 [file pharmaceutics-18-00301-s001.zip › Table S2.pdf]

Table S2 Calculated values of just suspended impeller speed and just suspended impeller tip speed for the different impeller geometries and clearances, equation 2 was used for the calculations, with Zwietering constants from [24] used. These constant equations can be seen in equations S1-S4.

**Impeller Geometry**	**Clearance (mm)**	**C/D**	N_{js} **(rps)**	I_{js} **(m/s)**
Flat Blade	18	0.200	2.408	0.378
	20	0.222	2.576	0.405
	25	0.278	2.971	0.467
	27	0.300	3.120	0.490
	30	0.333	3.338	0.524
Propeller	18	0.200	2.318	0.364
	20	0.222	2.400	0.377
	25	0.278	2.582	0.406
	27	0.300	2.649	0.416
	30	0.333	2.742	0.431
Rushton	18	0.200	2.897	0.455
	20	0.222	2.964	0.466
	25	0.278	3.112	0.489
	27	0.300	3.164	0.497
	30	0.333	3.238	0.509
Pitched Blade	18	0.200	2.414	0.379
	20	0.222	2.532	0.398
	25	0.278	2.803	0.440
	27	0.300	2.903	0.456
	30	0.333	3.045	0.478

Flat Blade impeller:

$$S = 13.98\frac{C}{T}^{0.639}\frac{H}{T}^{-0.055} \quad \text{(S1)}$$

Propeller:

$$S = 8.17\frac{C}{T}^{0.329}\frac{H}{T}^{-0.244} \quad \text{(S2)}$$

Rushton Turbine:

$$S = 8.54\frac{C}{T}^{0.218}\frac{H}{T}^{-0.248} \quad \text{(S3)}$$

Pitched Blade:

$$S = 10.42\frac{C}{T}^{0.455}\frac{H}{T}^{-0.107} \quad \text{(S4)}$$

Where: C is impeller clearance (m), H is liquid level (m), T is vessel diameter (m)
