# Supplementary figures and images for: A CFD and Experimental Investigation of the Influence of Flow Characteristics on Spherical Agglomeration
Source: Pharmaceutics. 2026 Feb 27;18(3):301. doi: 10.3390/pharmaceutics18030301 (PMC13029191; doi:10.3390/pharmaceutics18030301)

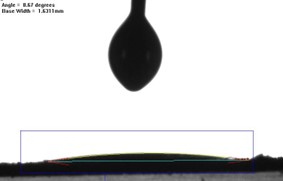

Supplement: Supplementary file 1 [file pharmaceutics-18-00301-s001.zip › Figure S1.tiff]

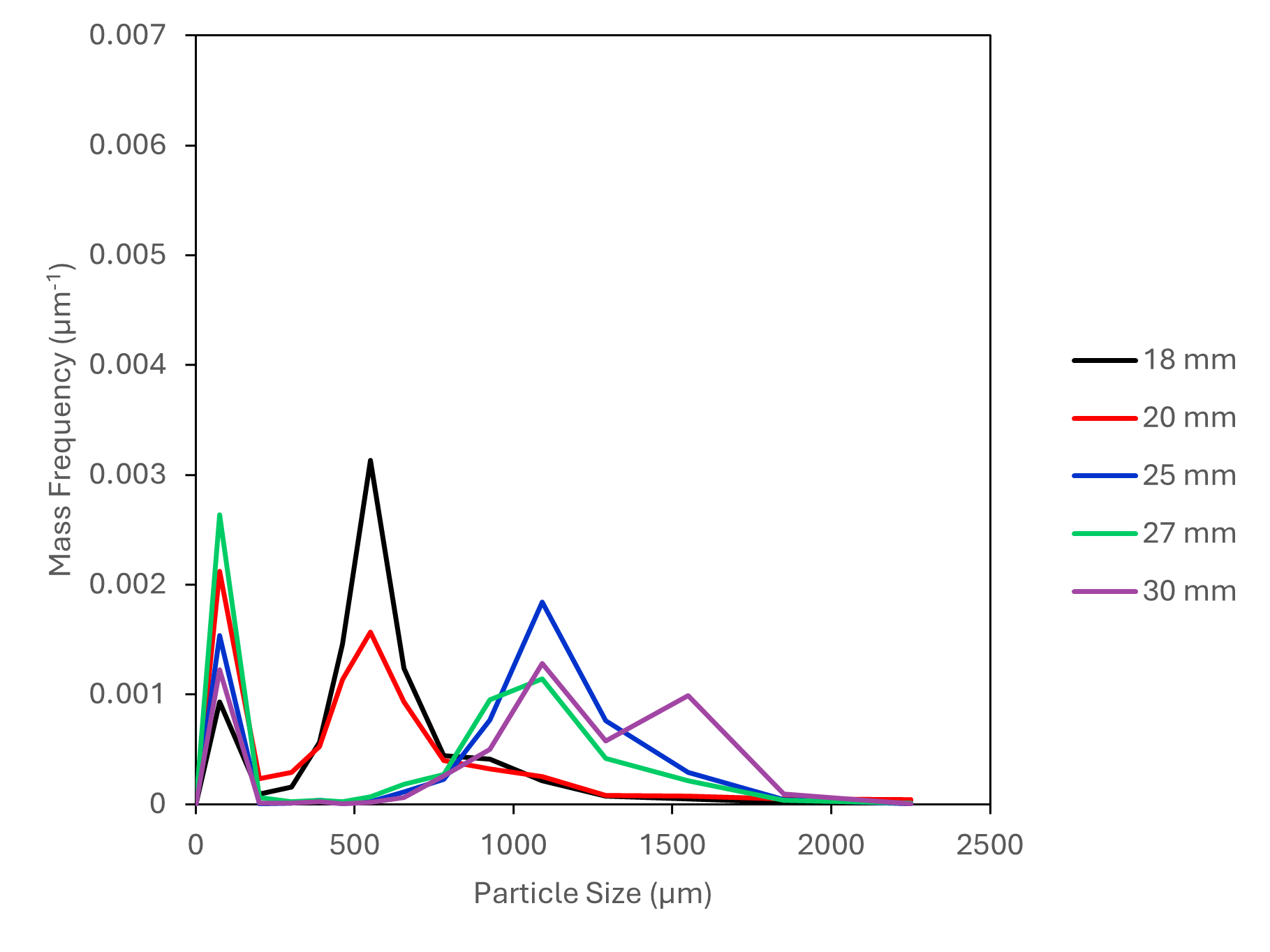

Supplement: Supplementary file 1 [file pharmaceutics-18-00301-s001.zip › Figure S10.tif]

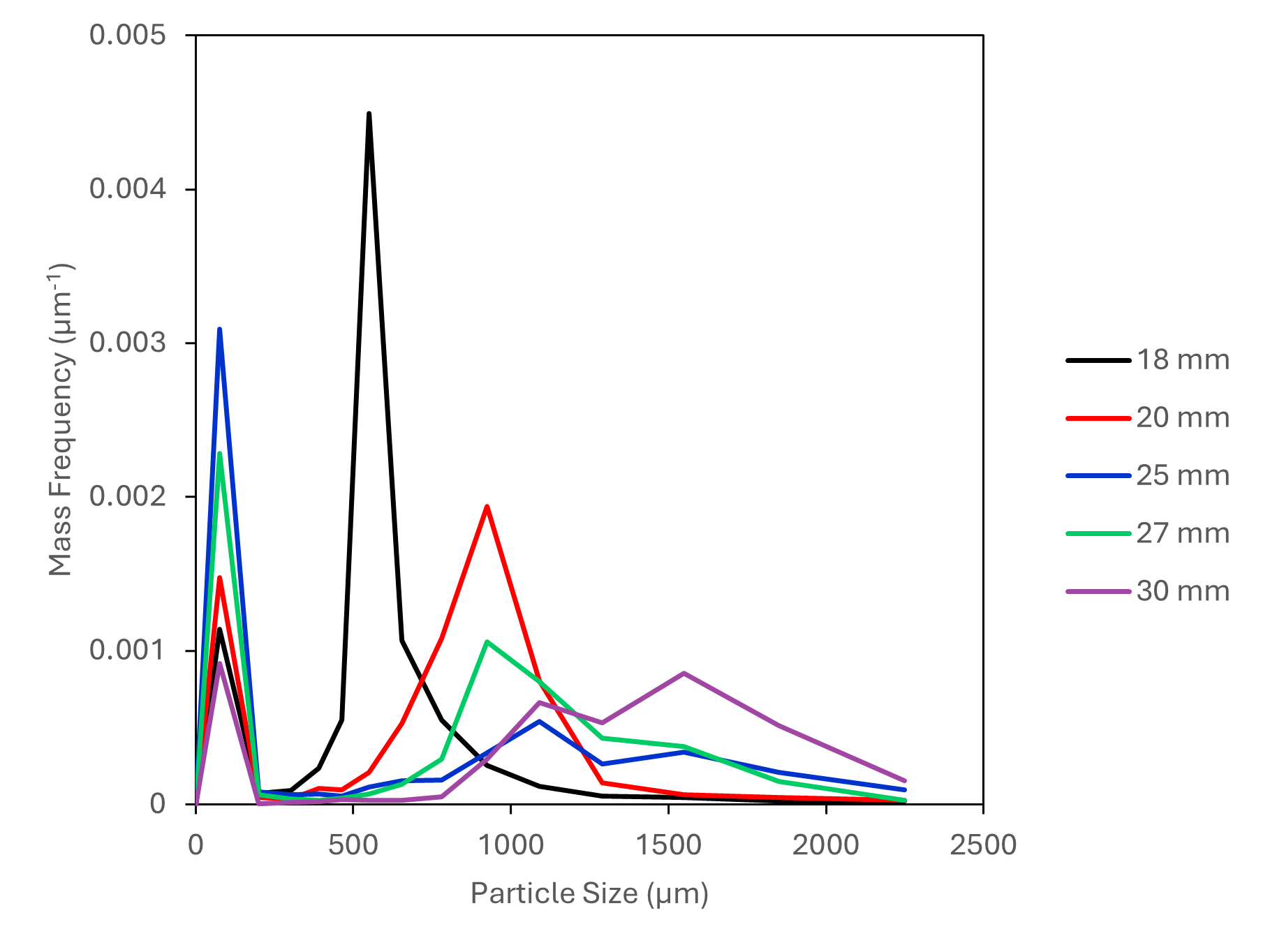

Supplement: Supplementary file 1 [file pharmaceutics-18-00301-s001.zip › Figure S11.tif]

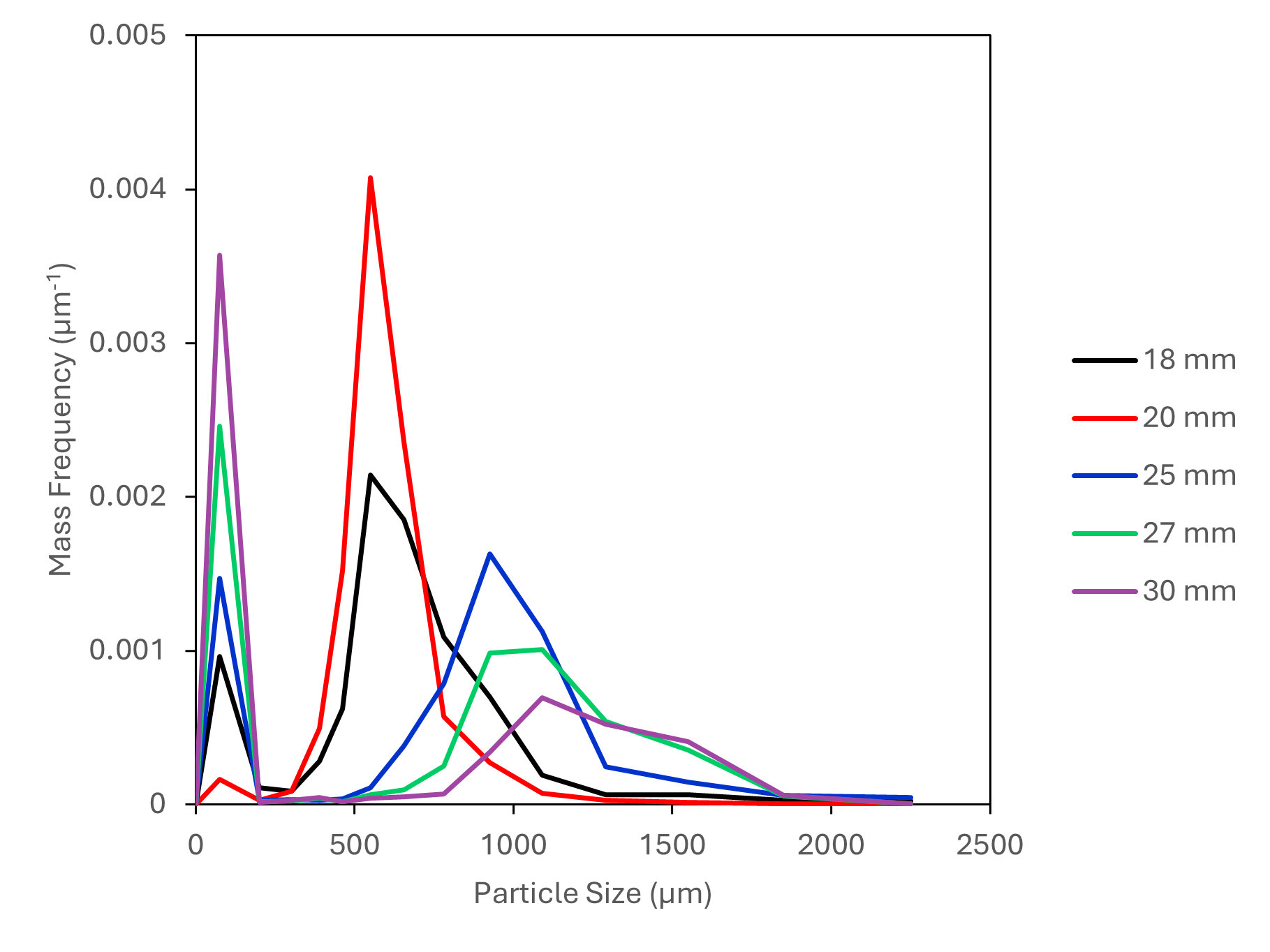

Supplement: Supplementary file 1 [file pharmaceutics-18-00301-s001.zip › Figure S12.tif]

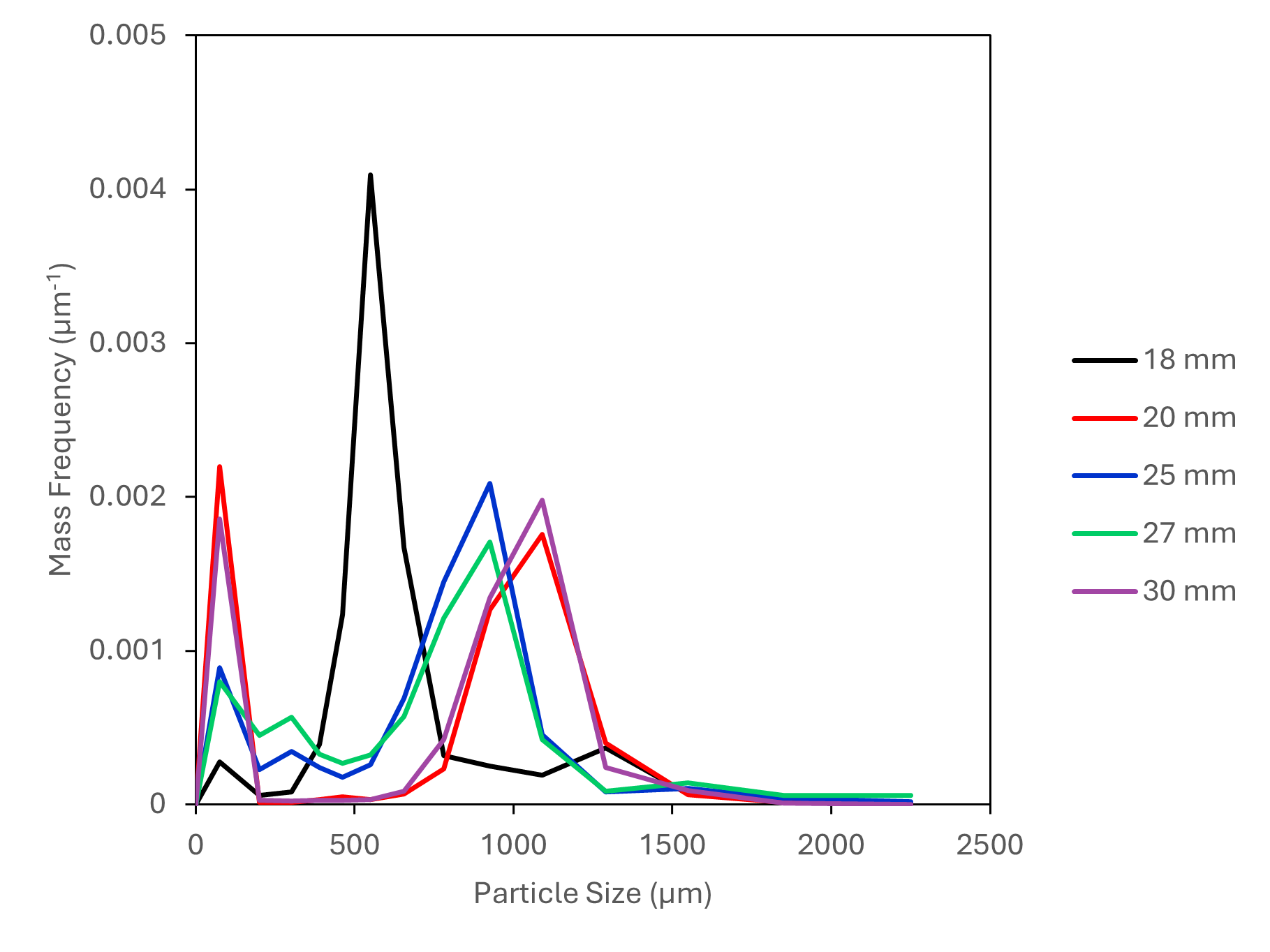

Supplement: Supplementary file 1 [file pharmaceutics-18-00301-s001.zip › Figure S13.tif]

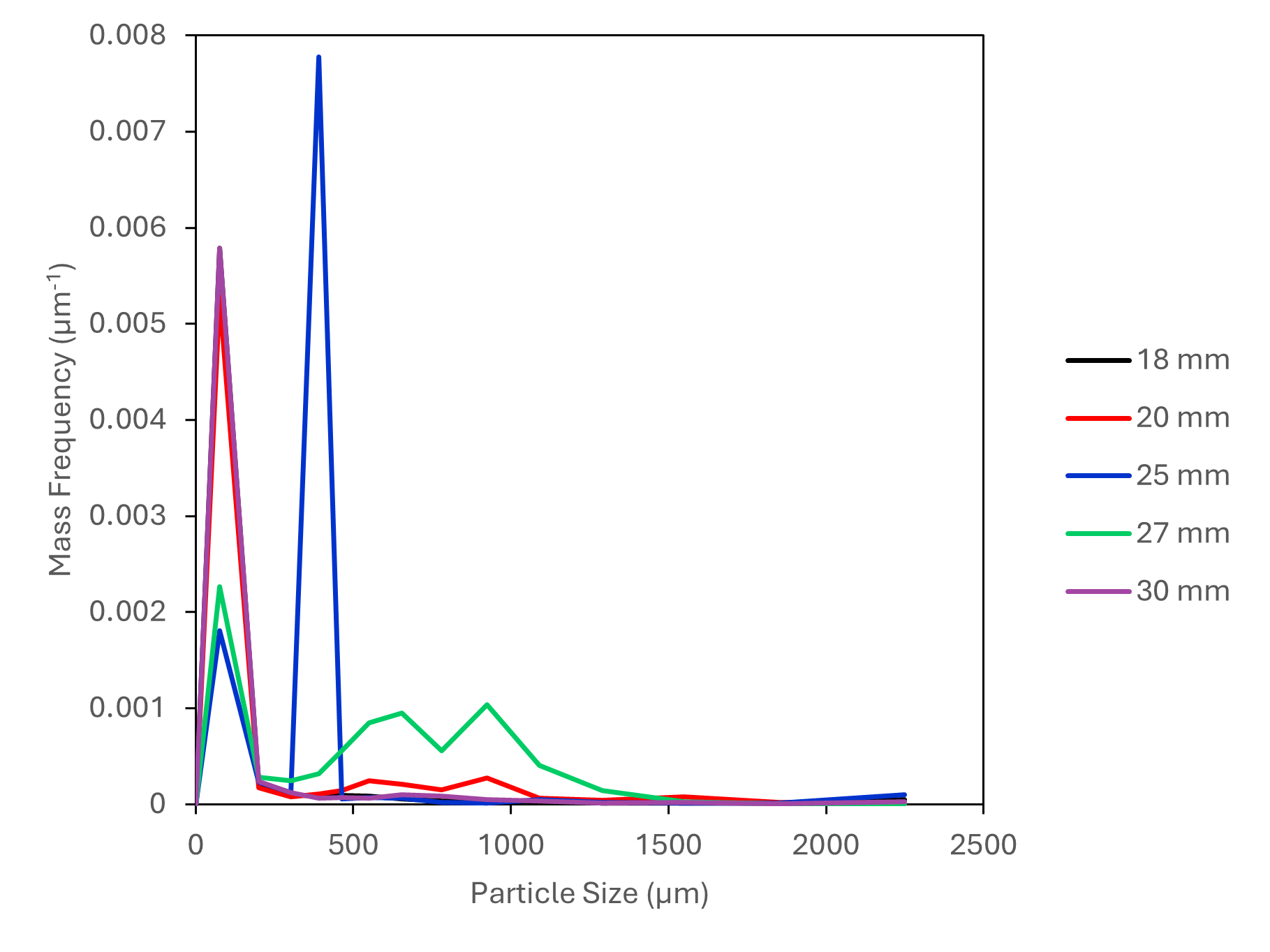

Supplement: Supplementary file 1 [file pharmaceutics-18-00301-s001.zip › Figure S14.tif]

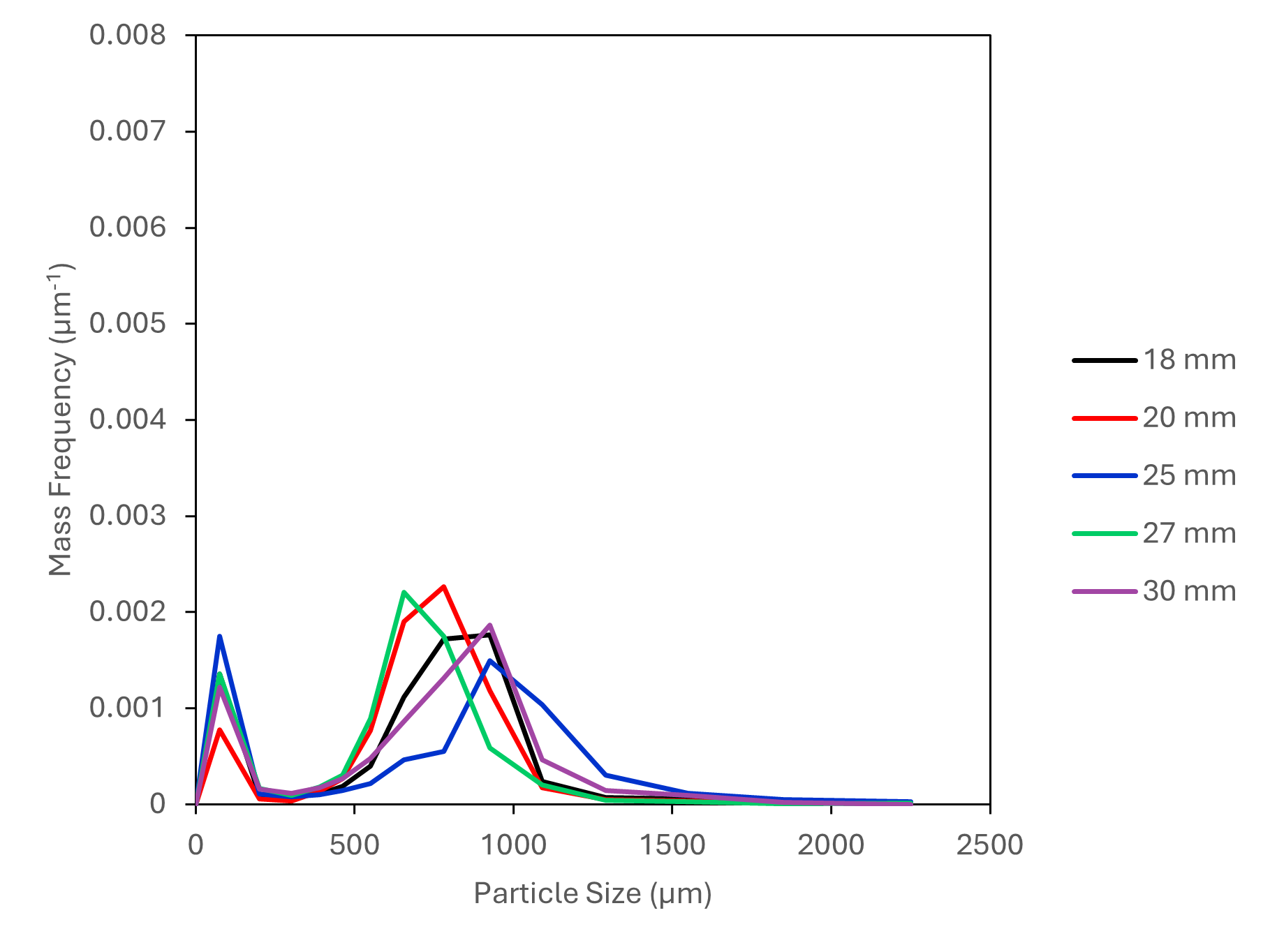

Supplement: Supplementary file 1 [file pharmaceutics-18-00301-s001.zip › Figure S15.tif]

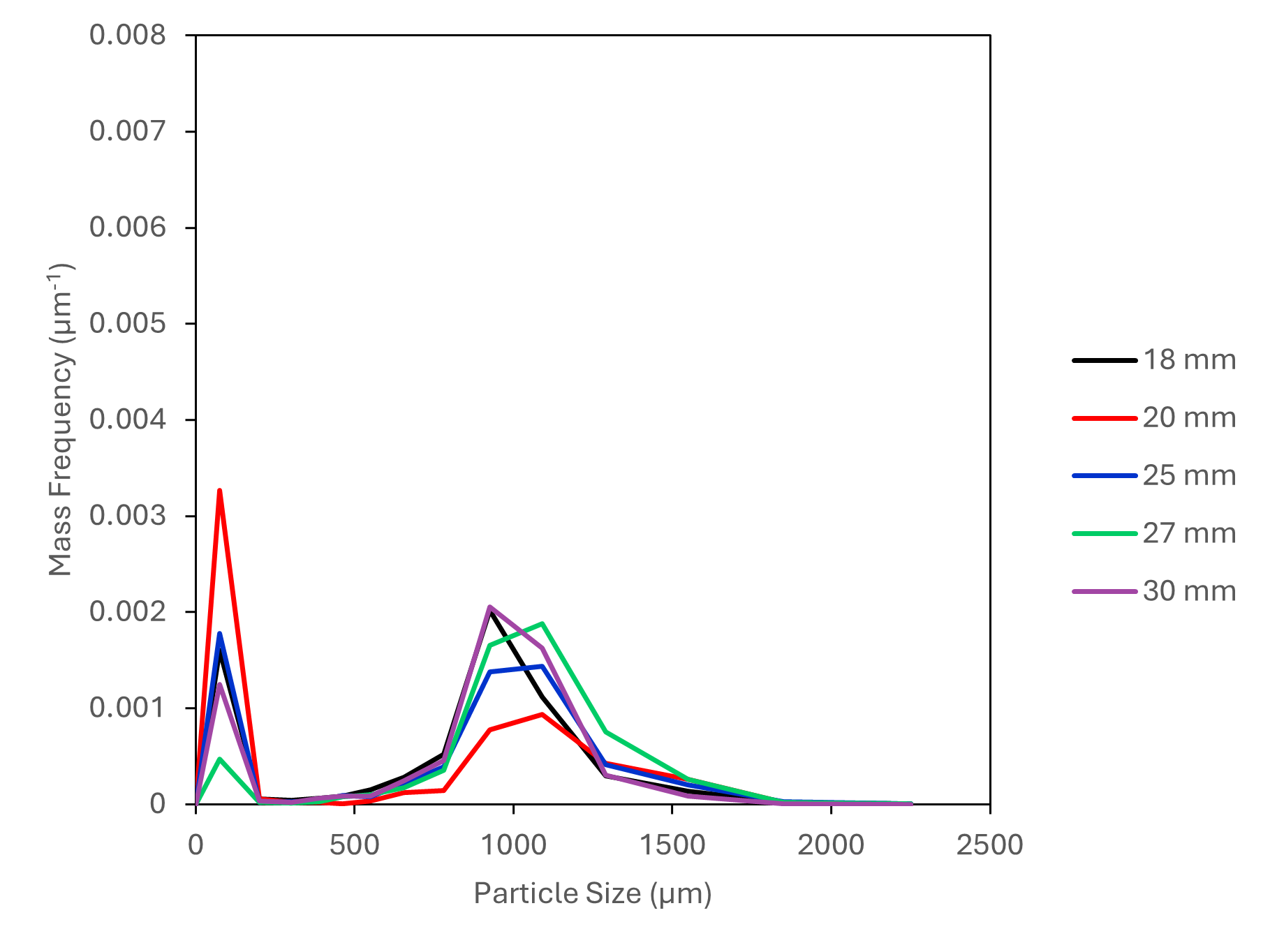

Supplement: Supplementary file 1 [file pharmaceutics-18-00301-s001.zip › Figure S16.tif]

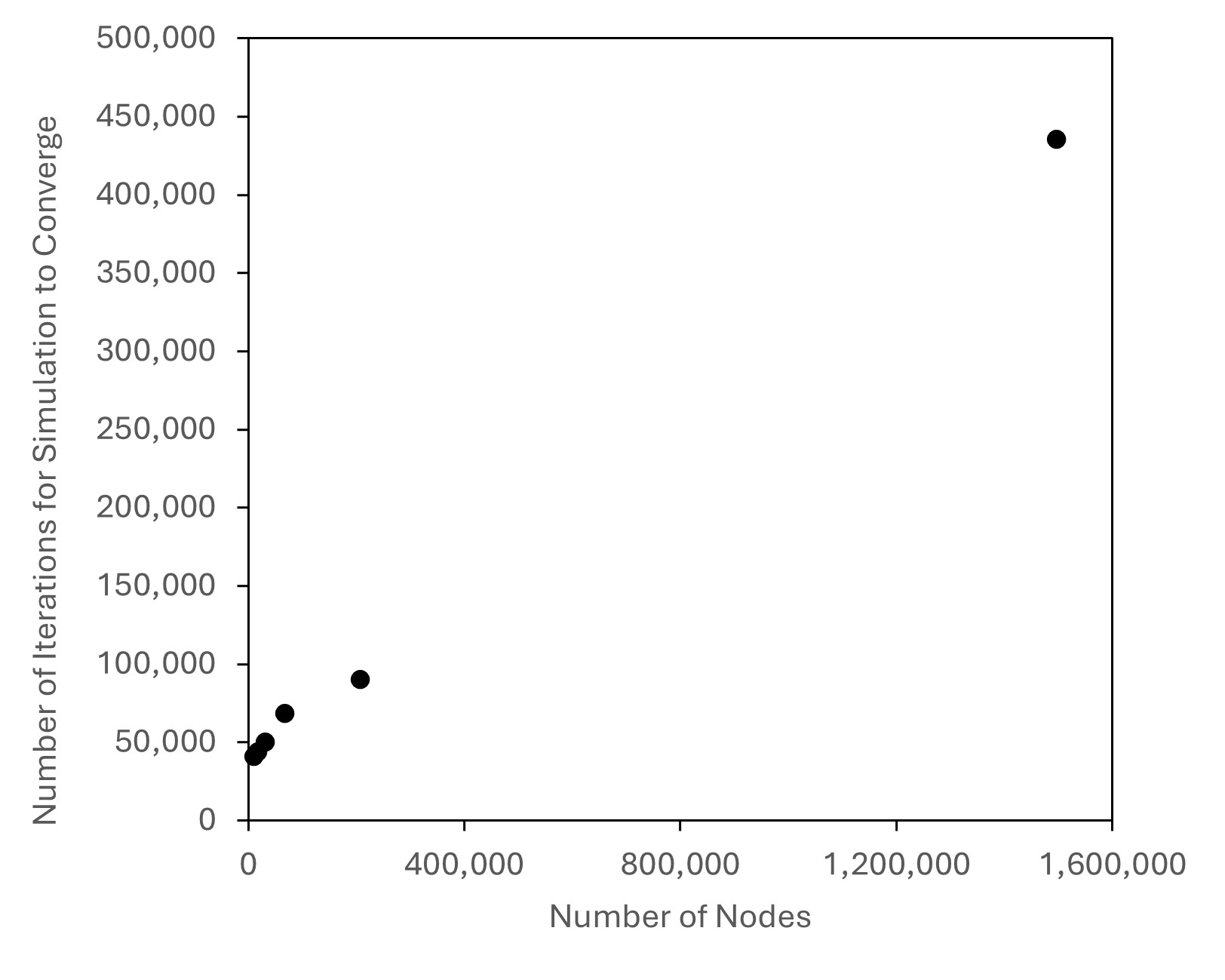

Supplement: Supplementary file 1 [file pharmaceutics-18-00301-s001.zip › Figure S2.tiff]

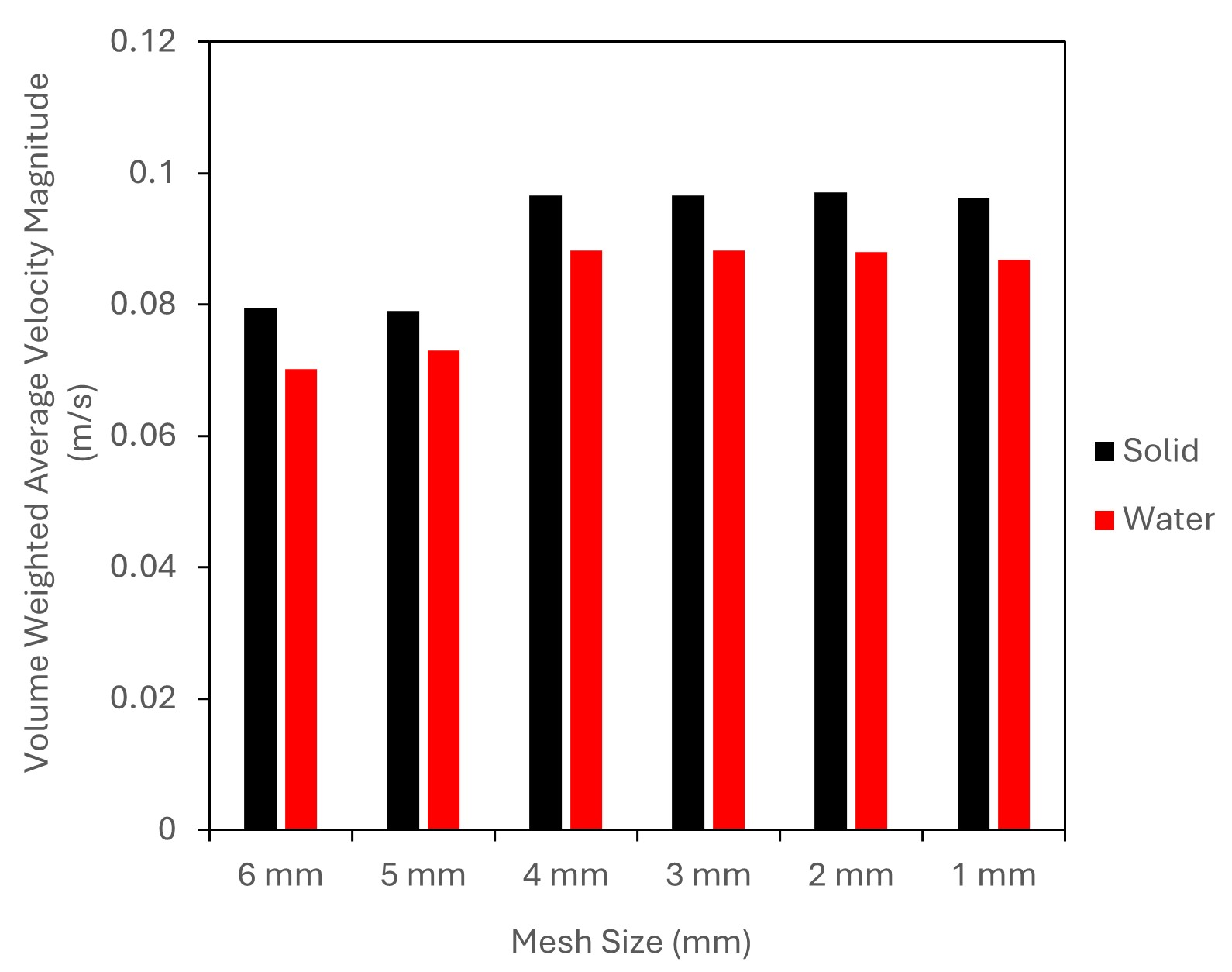

Supplement: Supplementary file 1 [file pharmaceutics-18-00301-s001.zip › Figure S3.tiff]

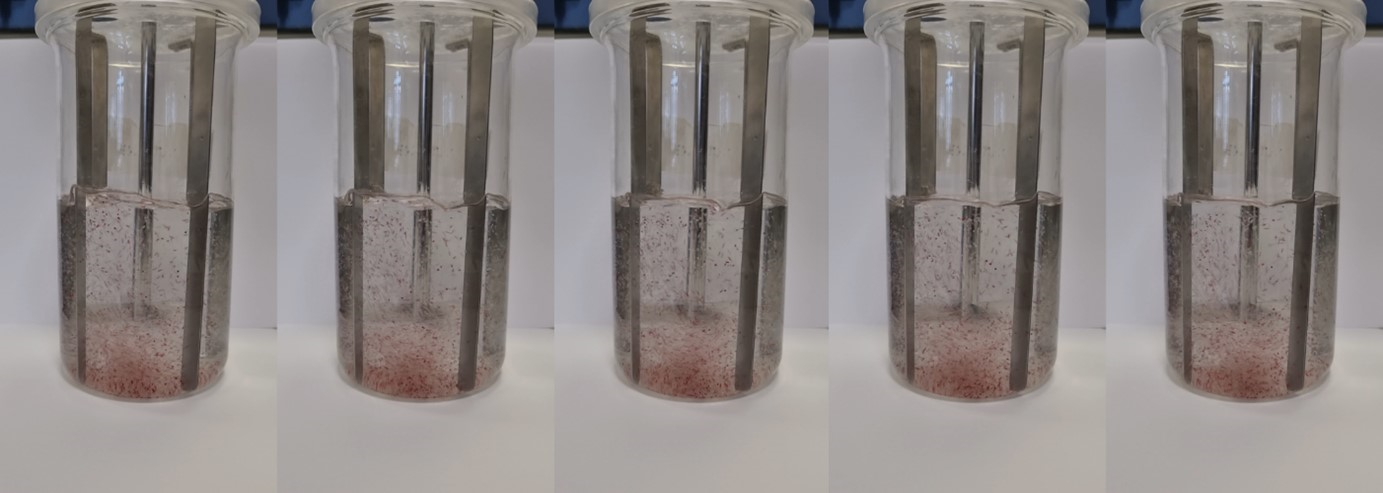

Supplement: Supplementary file 1 [file pharmaceutics-18-00301-s001.zip › Figure S4.tiff]

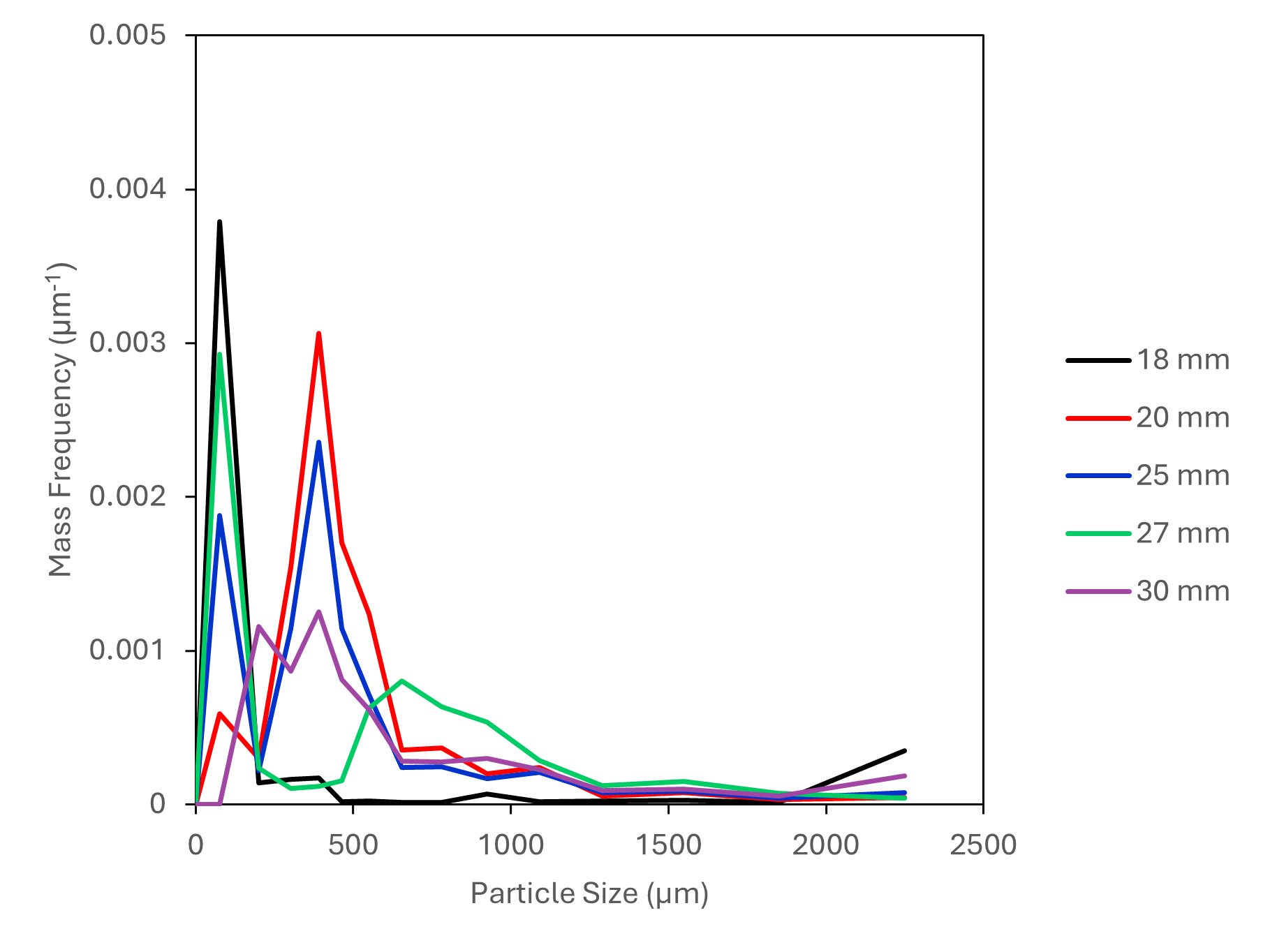

Supplement: Supplementary file 1 [file pharmaceutics-18-00301-s001.zip › Figure S5.tif]

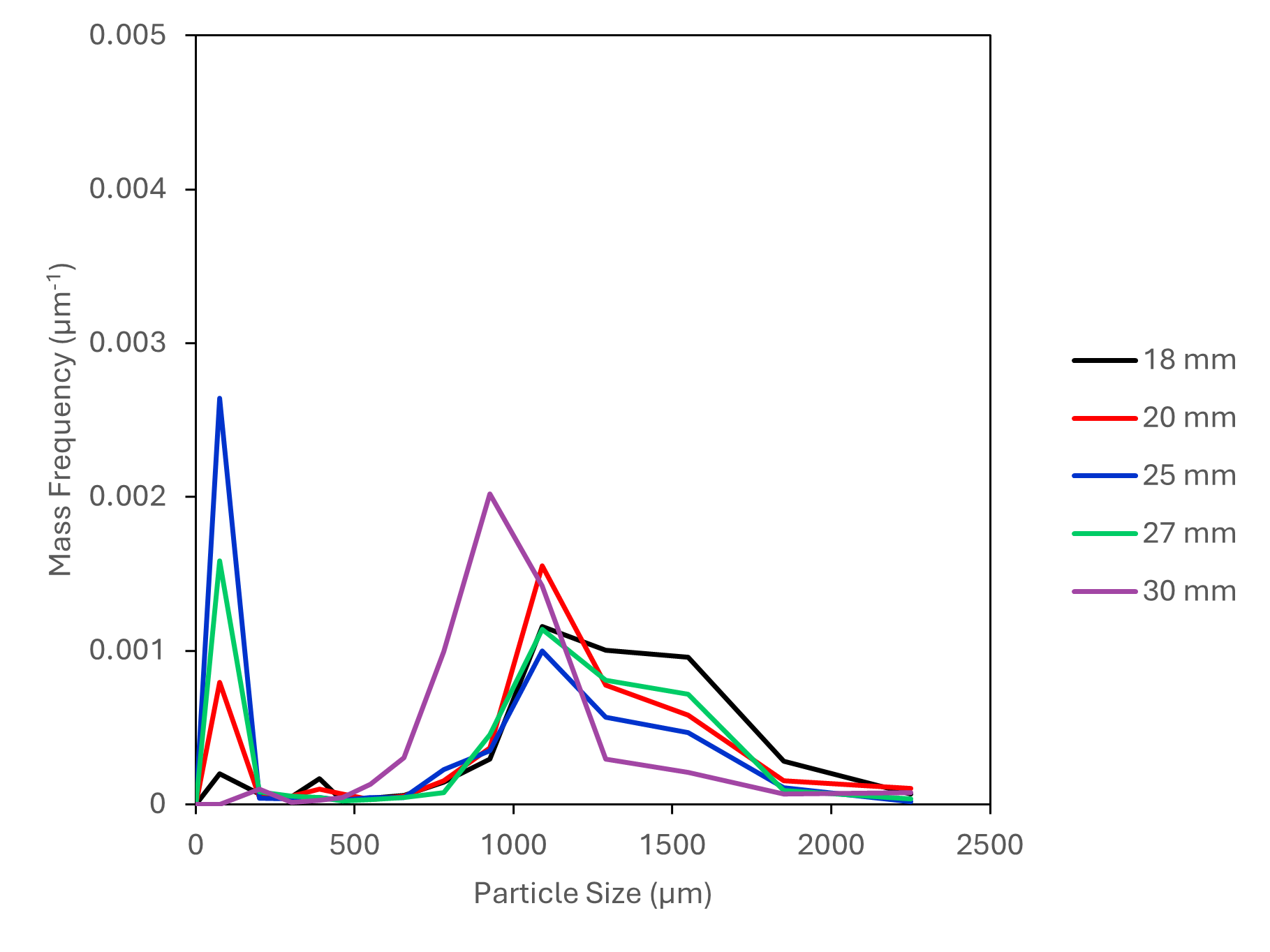

Supplement: Supplementary file 1 [file pharmaceutics-18-00301-s001.zip › Figure S6.tif]

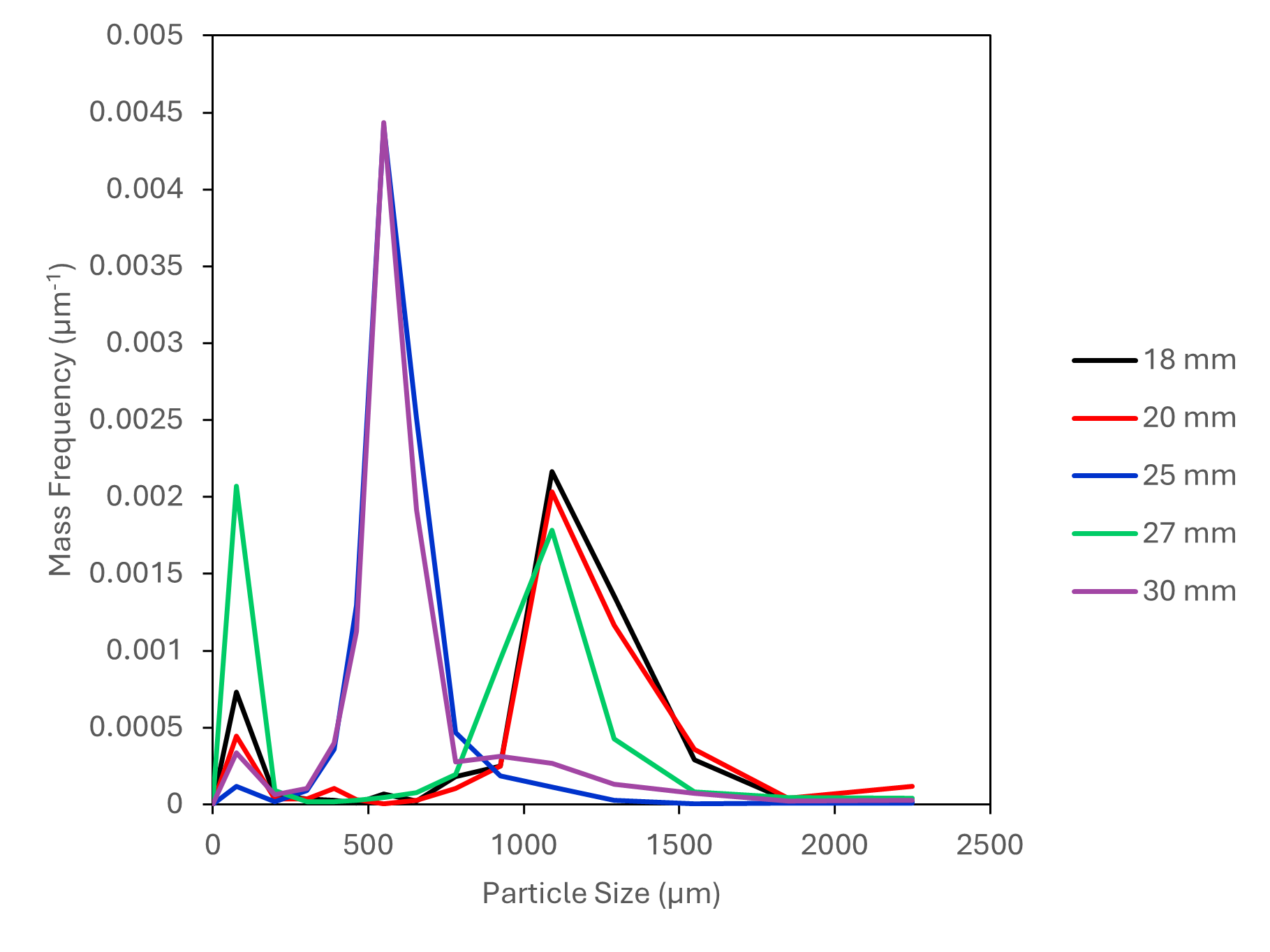

Supplement: Supplementary file 1 [file pharmaceutics-18-00301-s001.zip › Figure S7.tif]

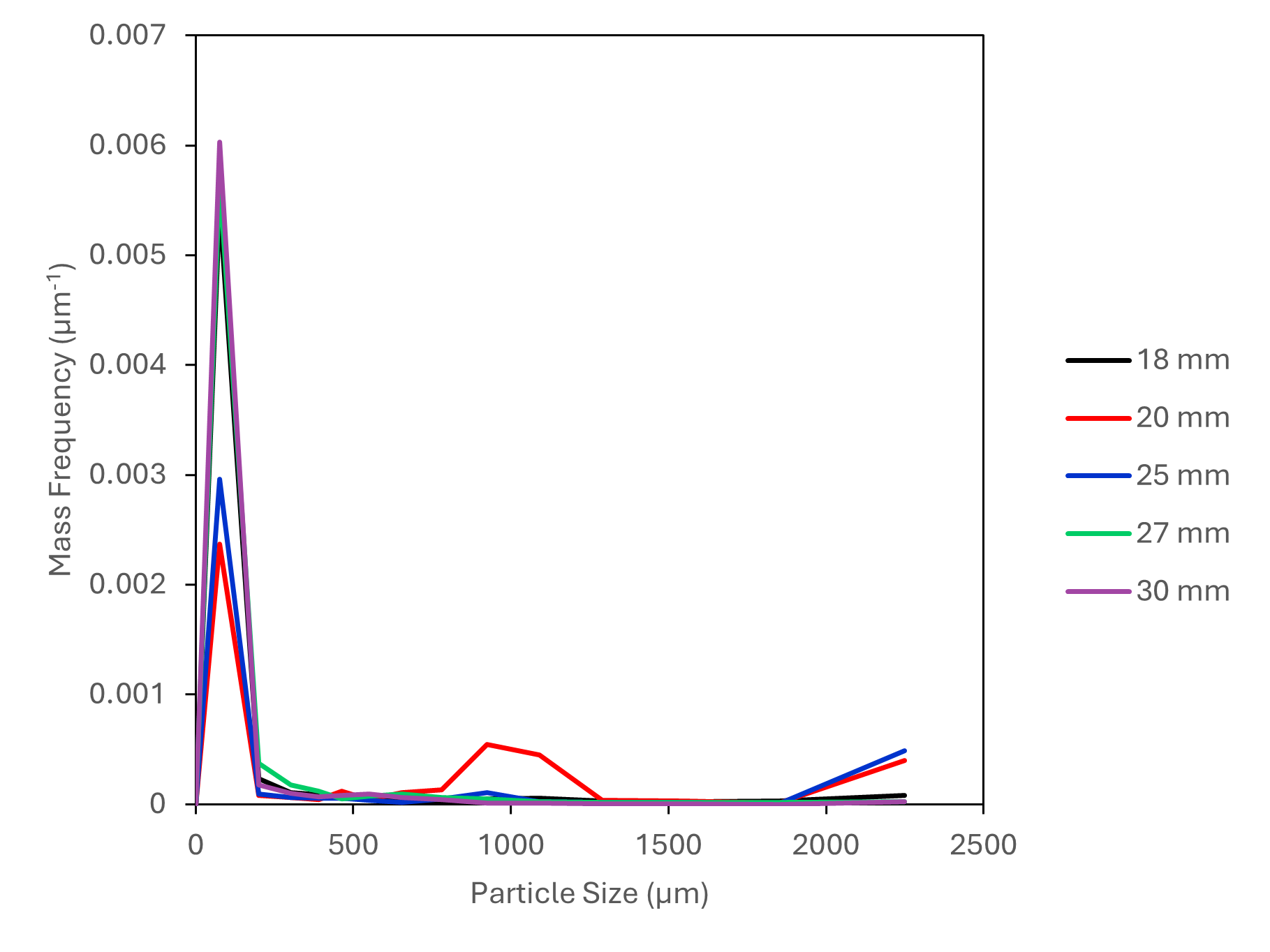

Supplement: Supplementary file 1 [file pharmaceutics-18-00301-s001.zip › Figure S8.tif]

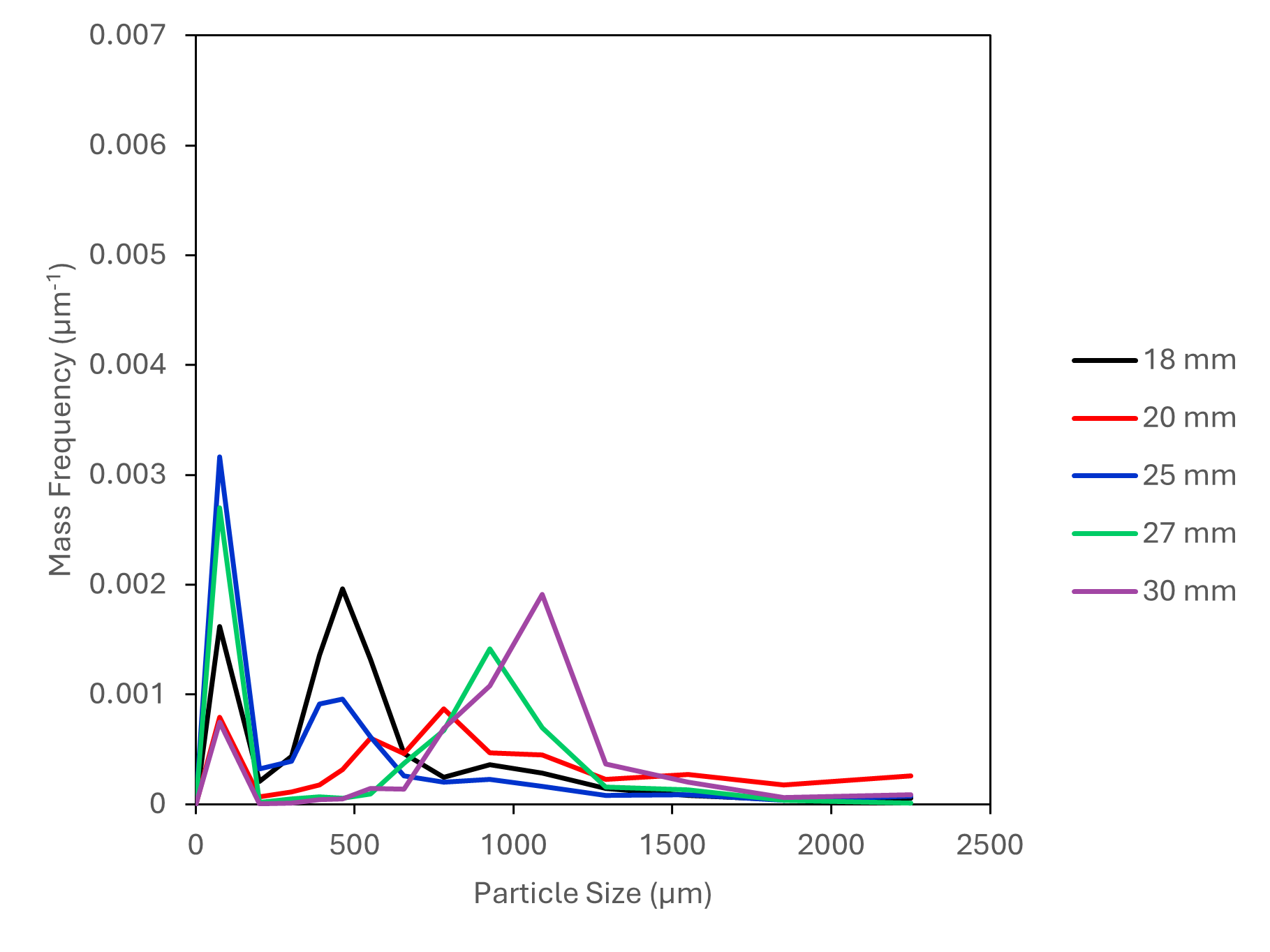

Supplement: Supplementary file 1 [file pharmaceutics-18-00301-s001.zip › Figure S9.tif]
